# Loss of connectin novex-3 leads to heart dysfunction associated with impaired cardiomyocyte proliferation and abnormal nuclear mechanics

**DOI:** 10.1038/s41598-024-64608-1

**Published:** 2024-06-14

**Authors:** Ken Hashimoto, Momoko Ohira, Aya Kodama, Misaki Kimoto, Mariko Inoue, Shigenobu Toné, Yuu Usui, Akira Hanashima, Takato Goto, Yuhei Ogura, Yoshihiro Ujihara, Satoshi Mohri

**Affiliations:** 1 First Department of Physiology, Kawasaki Medical School, Kurashiki, 701-0192 Japan; 2 Central Research Institute, Kawasaki Medical School, Kurashiki, 701-0192 Japan; 3 Laboratory of Molecular Developmental Biology, Graduate School of Science and Engineering, Tokyo Denki University, Hatoyama, Saitama 350-0394 Japan; 4 Department of Electrical and Mechanical Engineering, Nagoya Institute of Technology, Nagoya, 466-8555 Japan

**Keywords:** Cardiology, Cardiovascular biology, Cardiac regeneration

## Abstract

Connectin (also known as titin) is a giant striated muscle protein that functions as a molecular spring by providing elasticity to the sarcomere. Novex-3 is a short splice variant of connectin whose physiological function remains unknown. We have recently demonstrated using in vitro analyses that in addition to sarcomere expression, novex-3 was also expressed in cardiomyocyte nuclei exclusively during fetal life, where it provides elasticity/compliance to cardiomyocyte nuclei and promotes cardiomyocyte proliferation in the fetus, suggesting a non-sarcomeric function. Here, we analyzed novex-3 knockout mice to assess the involvement of this function in cardiac pathophysiology in vivo. Deficiency of novex-3 compromised fetal cardiomyocyte proliferation and induced the enlargement of individual cardiomyocytes in neonates. In adults, novex-3 deficiency resulted in chamber dilation and systolic dysfunction, associated with Ca^2+^ dysregulation, resulting in a reduced life span. Mechanistic analyses revealed a possible association between impaired proliferation and abnormal nuclear mechanics, including stiffer nuclei positioned peripherally with stabilized circumnuclear microtubules in knockout cardiomyocytes. Although the underlying causal relationships were not fully elucidated, these data show that novex-3 has a vital non-sarcomeric function in cardiac pathophysiology and serves as an early contributor to cardiomyocyte proliferation.

## Introduction

Coordinated regulation of the proliferation and differentiation of cardiomyocytes (CMs) are essential for normal cardiac development. CMs are highly proliferative during early fetal life but show limited differentiation, yielding a large number of immature CMs. This hyperplastic state switches to a hypertrophic state around birth when the CMs permanently exit the cell cycle and become committed exclusively to differentiation, resulting in mature specialized CMs that work for the entire lifespan of the organism^1^. This fine-tuned regulation is indispensable, because perturbing it in rodent models as a means of therapeutic manipulation often has unwanted consequences, such as heart failure^2–4^.

Connectin (also known as titin, *Ttn*) is the giant striated muscle protein (3–4 MDa) that spans from the Z-disk, I-band, and A-band to the M-band of the sarcomere^5,6^. It has received great attention because its mutations are the leading cause of familial dilated cardiomyopathy^7^. Three major isoforms (N2BA, N2B, and N2A) are found, as well as numerous variants, including shorter fragments that are produced through complex alternative splicing^7^. Generally, connectin functions as a molecular spring by providing elasticity to the sarcomere^5^, but non-sarcomeric functions of connectin have long been proposed. For example, connectin homologues in several species, including vertebrates and invertebrates, are found in the nucleus and provide elasticity/compliance to nuclear structures in non-muscle and muscle cells^8–12^. Some of these homologues have also been proposed as cell cycle promoters^10,12–15^; however, no role for these connectin homologues in cardiac pathophysiology has been established to date.

**Figure 1:**
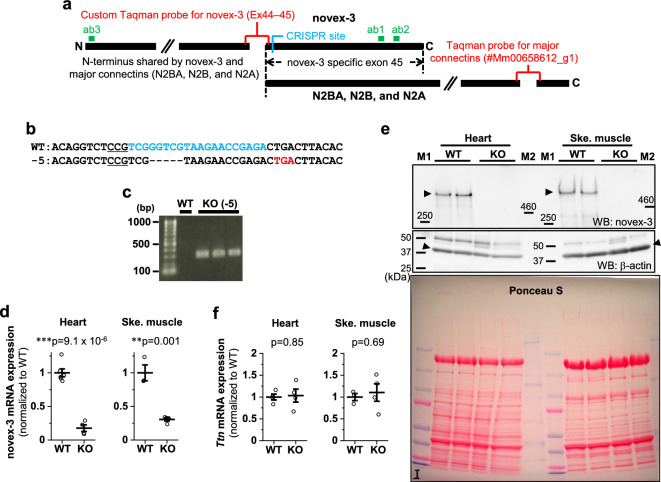

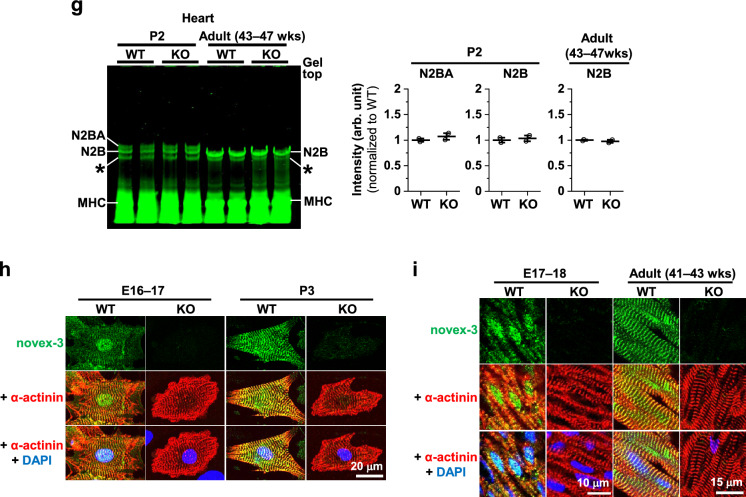
Generation of novex-3 KO mice. (**a**) Schematic diagram of connectin (titin) isoforms (not drawn to scale). Novex-3 (ENSMUST00000099980.10) shares the N-terminal region with the major connectin isoforms (N2BA and N2B in cardiac muscles and N2A in skeletal muscles), but has an alternative termination signal encoded by a large novex-3-specific exon 45 that is not utilized in the major connectins. We targeted the N-terminus of this exon by CRISPR/Cas9-mediated genome editing (shown in blue). Taqman probe for novex-3 was custom designed at the boundary between exon 44 and 45 not to affect the CRISPR cleavage site (shown in red). Taqman probe Mm00658612_g1 (Thermo-Fisher) targeting the C-terminal region of full-length connectin, but not novex-3, was used for detecting major isoforms (*Ttn*, shown in red). Two polyclonal antibodies targeting the novex-3-specific exon were used (designated as ab1 and ab2, shown in green). One was from Myomedix #TTN-2 (ab1) and mainly used for Western blot. The other (ab2) was raised against a synthetic peptide EKDVKEFEKQVKIV [AA 5222–5235] of mouse novex-3 and mainly used for immunofluorescence. The additional antibody directed against the N-terminal shared region (ab3)^44^ were used for detecting both novex-3 and full-length major connectin on the same blot. (**b**) Genomic sequence of novex-3 KO mice lines with 5 bp deletion (− 5) generated by CRISPR as compared to WT sequence. gRNA sequence in blue, PAM sequence underlined, a novel stop codon in red, deleted base is depicted as “–”. (**c**) Representative genotyping using tail DNA for 1 WT and 3 KO mice at 3 weeks of age. Primer sequence: 5′-GAACTCTTTGAGGGGGAAGC-3′ and 5′-CTCGGTTCTTACGACGGA-3′. (**d**) qPCR analysis of novex-3 transcripts in hearts and skeletal muscles from WT/KO mice using primers targeting exon 44–45 shown in (**a**). Data are shown as normalized to WT. n = 3–6 mice per group (6–19 weeks). **p < 0.01, ***p < 0.001 as compared to WT by Student’s two-tailed unpaired t-test. Error bar = SEM. (**e**) Western blot for novex-3 proteins (arrowhead) in hearts and skeletal muscles from WT/KO mice (15–36W) using an antibody against the novex-3-specific region (ab1 shown in (**a**)). The representative blot for two mice in each condition is shown. The blot for β-actin (marked as arrowhead) and Ponceau S staining of the membrane were done as a loading control. M1: size marker (10–250 kDa), M2: size marker (30–460 kDa). Original blots are presented in Supplementary Fig. 6. (**f**) qPCR analysis using primers detecting major connectin isoforms (*Ttn*; N2BA and N2B in cardiac muscles and N2A in skeletal muscles), but not novex-3 (shown in (**a**)) from WT/KO mice. Data are shown as normalized to WT. n = 3–4 mice per group (6–19 weeks). Exact *p* values as compared to WT by Student’s two-tailed unpaired t-test are shown. Error bar = SEM. (**g**) SDS-vertical agarose gel electrophoresis detecting major connectin protein isoforms expressed in the heart (N2BA and N2B) from WT/KO mice at the indicated age (43–47 weeks for adult mice). A representative gel for two mice in each condition is shown. The intensity of each band was quantified. *: a mixed band of recently identified Cronos isoform^7,54^ and a proteolytic degradation product of full connectin molecule termed T2/β-connectin^7,55^. MHC: myosin heavy chain. (**h,i**) Immunofluorescence of novex-3 using an antibody ab2 shown in (**a**), costained with sarcomeric α-actinin (as a CM marker) and DAPI in dissociated CMs (**h**) and in heart tissue sections (**i**) from WT/KO mice at the indicated age (E: embryonic day, P: postnatal day, 41–43 weeks for adult mice).

Novex-3, as a shorter 650 kDa connectin variant, is one of these homologues. It shares the N-terminal region with the major connectins, including the nuclear localization signal (NLS)^13^, but has an alternative termination signal encoded by a large unique exon that is not utilized in the major connectins (novex-3-specific exon) (Fig. 1a)^16^. The primary structure of novex-3 is well conserved among vertebrates, including humans, mice, cows, chickens, frogs (*Xenopus laevis*), and zebrafish^17–19^, suggesting that it plays an important role in muscle pathophysiology. However, since its discovery in 2001^16^, the physiological function of novex-3 has remained enigmatic. One study showed that novex-3 integrates into the Z-disk lattice and interacts with a myofibrillar protein obscurin to form an elastic Z-disc–to–I-band linking system in the sarcomere^16,20^ (although a later study failed to detect this novex-3-obscurin interaction^21^). A second study showed that the prominent expression of the novex-3 homologue in early *Xenopus laevis* embryos later declined at the tadpole stages^17^.

Our recent in vitro analyses demonstrated that in addition to sarcomere, CM nuclei express novex-3 exclusively during fetal life, but cease its expression soon after birth in mice. Nuclear novex-3 has a unique function, as it provides elasticity/compliance to the CM nuclei, and contributes to CM proliferation in the fetus^12^, suggesting a non-sarcomeric function. The aims of the present study are therefore (1) to test whether this non-sarcomeric function of novex-3 is recapitulated in vivo using novex-3 knockout (KO) mice, and (2) to assess how this function is involved in cardiac pathophysiology. We propose that this connectin variant has a non-sarcomeric function, and that it serves as an early contributor to CM proliferation by providing elasticity/compliance to the CM nuclei.

## Results

### Generation of novex-3 KO mice

Because novex-3 shares the N-terminal region with major connectin isoforms (N2BA, N2B, and N2A), we targeted the N-terminus of the novex-3-specific exon by CRISPR/Cas9-mediated genome editing (Fig. 1a), which produced mouse lines with a 5 bp deletion causing a frameshift predicted to result in a novel stop codon (Fig. 1b, see methods for details). Genotypic PCR confirmed this frameshift effect (Fig. 1c). A complete deficiency of novex-3 protein was confirmed in cardiac and skeletal muscles using an antibody against the novex-3-specific region (ab1 in Fig. 1a), though residual expression at the transcript level was detected for unknown reasons (Fig. 1d, e and Supplementary Fig. 1a.). qPCR analyses revealed no effect on the major connectin isoforms (N2BA and N2B in cardiac muscles and N2A in skeletal muscles) (Fig. 1f), using primers targeting the connectin C-terminal region, but not novex-3 (Fig. 1a). This was confirmed at the protein level by SDS-vertical agarose gel electrophoresis of heart homogenates (Fig. 1g). Additional Western analysis using an antibody against the N-terminal shared region (ab3 in Fig. 1a), which detected both novex-3 and full-length major connectin on the same blot, confirmed the absence of the former and unaltered expression of the latter in cardiac and skeletal muscles from KO mice (Supplementary Fig. 1b, c). In wildtype (WT) mice, the typical sarcomeric distribution, as well as nuclear enrichment specifically during fetal life^12^, were confirmed in dissociated CMs (Fig. 1h) and in heart tissue sections (Fig. 1i). These novex-3 signals were never detected in the novex-3-deficient mice. This pattern of expression in WT mice was further corroborated in dissociated CMs exogenously expressing full-length novex-3 protein (Supplementary Fig. 1d) and in Western blot analysis of heart samples (Supplementary Fig. 1e, f). We used this line with a 5 bp deletion as a KO mouse line in subsequent experiments.

### Impaired CM proliferation during early fetal life in novex-3 KO mice

**Figure 2:**
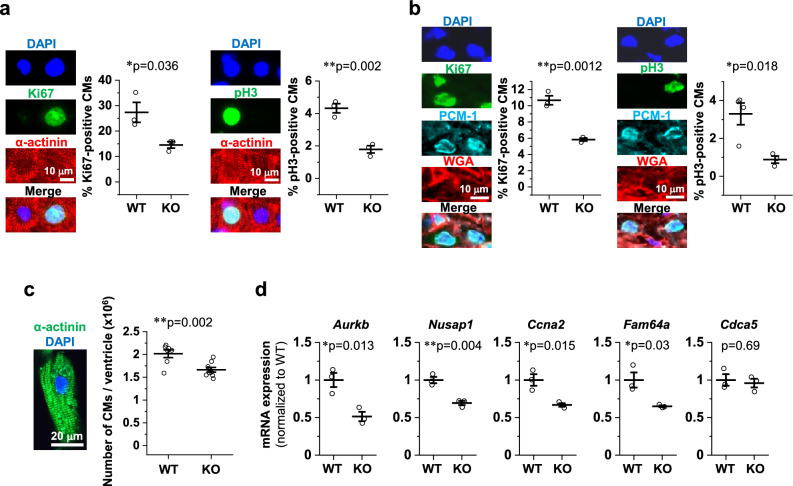

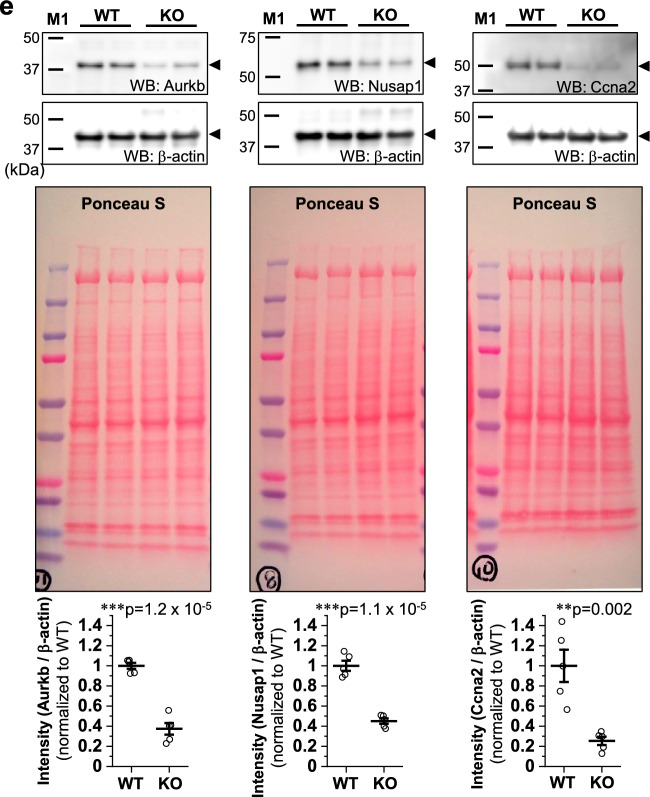

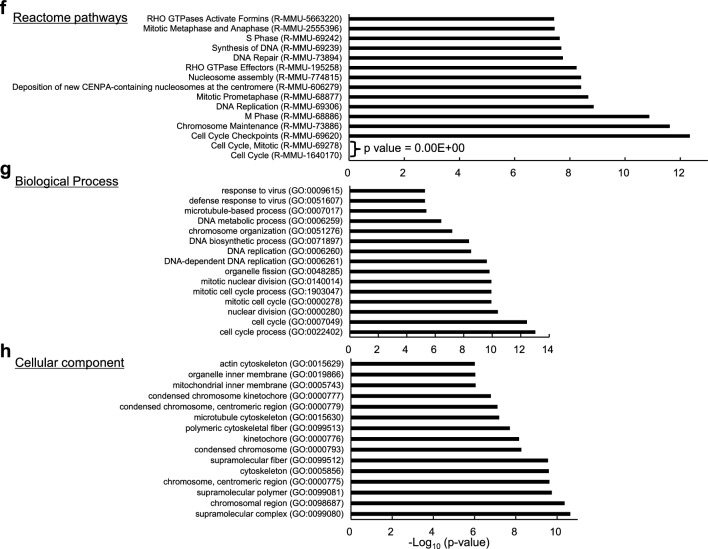

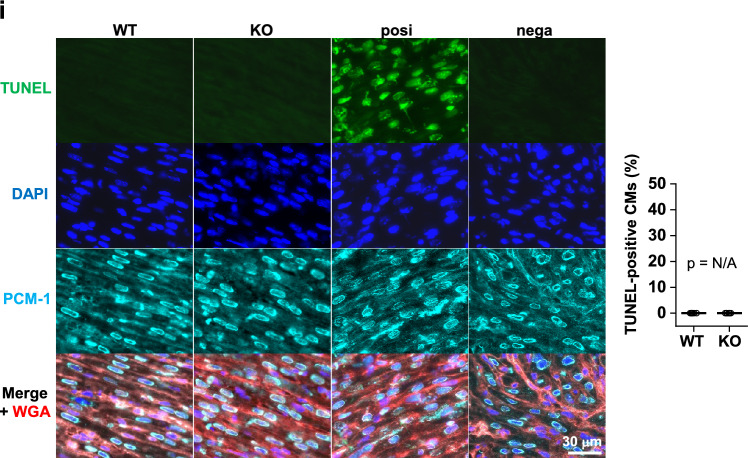
Impaired CM proliferation during early fetal life in novex-3 KO mice. (**a**) Immunofluorescence stainings for Ki67 (left) and phospho-histone H3 (pH3) (right), costained with sarcomeric α-actinin (as a CM marker) and DAPI in E12–E13 dissociated CM cultures from WT/KO mice. The percentage of Ki67-positive (left) and pH3-positive (right) CMs was quantified. n = 3 independent experiments. In each experiment, at least 500 CMs were counted. *p < 0.05 and **p < 0.01 as compared to WT by Student’s two-tailed unpaired t-test. Error bar = SEM. (**b**) Immunofluorescence stainings for Ki67 (left) and pH3 (right), costained with PCM-1 (as a CM marker), wheat germ agglutinin (WGA: to delineate cell borders), and DAPI in P0 heart tissue sections from WT/KO mice. The percentage of Ki67-positive (left) and pH3-positive (right) CMs was quantified. n = 3–4 mice per group. At least 500 CMs were counted from each mouse. *p < 0.05 and **p < 0.01 as compared to WT by Student’s two-tailed unpaired t-test. Error bar = SEM. (**c**) Number of CMs per ventricle in WT/KO mice at P13–P14 evaluated by the fixation digestion method. Representative image of a dissociated CM stained with α-actinin and DAPI is shown. n = 7–9 mice per group. **p < 0.01 as compared to WT by Student’s two-tailed unpaired t-test. Error bar = SEM. (**d**) qPCR analysis of cell cycle promoting gene transcripts in P0 hearts from WT/KO mice. Data are shown as normalized to WT. n = 3 mice per group. *p < 0.05 and **p < 0.01 as compared to WT by Student’s two-tailed unpaired t-test. Error bar = SEM. (**e**) Western blot for cell cycle promoting genes in P0–2 hearts from WT/KO mice. The representative blot for two mice in each condition is shown. The blot for β-actin (marked as arrowhead) and Ponceau S staining of the membrane were done as a loading control. M1: size marker (10–250 kDa). Original blots are presented in Supplementary Fig. 6. Densitometric analysis with β-actin as a reference control is shown as normalized to WT. n = 5 mice per group. **p < 0.01 and ***p < 0.001 as compared to WT by Student’s two-tailed unpaired t-test. Error bar = SEM. (**f**–**h**) Based on RNA-seq data comparing gene expressions in WT versus KO mice hearts at P1 (n = 2 mice per group), statistical enrichment test was performed using Panther at http://www.pantherdb.org/^51^. The top 15 rank order of potency for *p* value for reactome pathways (**f**), biological processes (**g**), and cellular components (**h**) are shown. See methods for details. (**i**) TdT-mediated dUTP Nick End Labeling (TUNEL) assay co-immunostained with PCM-1 (as a CM marker), wheat germ agglutinin (WGA: to delineate cell border) and DAPI in P6–P7 heart tissue sections from WT/KO mice. Positive controls (posi) were treated with DNase I to generate DNA breaks. Negative controls (nega) were processed with terminal deoxynucleotidyl transferase (TdT) enzyme omitted from the labeling reaction. The percentage of TUNEL-positive CMs was quantified from the total CMs that were identified by PCM-1 and DAPI signals. n = 6 mice per group. In each mouse, at least 500 CMs were counted. Student’s two-tailed unpaired t-test were not applicable (N/A) because all data were zero. Error bar = SEM.

We previously demonstrated a role for novex-3 in promoting CM proliferation in fetuses^12^. In the present study, we first assessed cell cycle activity in dissociated fetal CMs (embryonic day E12–13) by staining for Ki67, a cell cycle marker, and phospho-histone H3 (pH3), a mitosis marker. The numbers of both Ki67-positive and pH3-positive CMs were significantly lower in the KO mice than in the WT mice (Fig. 2a). Similar results were obtained for heart sections analyzed at postnatal day 0 (P0) (Fig. 2b). Accordingly, the total CM count per ventricle was decreased in KO mice at P13–14 (Fig. 2c). Subsequent qPCR and Western analysis revealed depressed expression of several cell cycle genes^4^ in KO hearts at P0–2 (Fig. 2d, e). Statistical enrichment analysis of RNA-seq data from P1 heart samples (WT vs. KO) indicated enrichment of the cell cycle pathway (Fig. 2f–h, see methods and Supplementary Data 1 for details). Meanwhile, our TdT-mediated dUTP Nick End Labeling (TUNEL) assay demonstrated no apoptosis in either WT or KO CMs at P6–7 (Fig. 2i), excluding the possibility that the reduced CM number in KO mice (Fig. 2c) was attributed to increased apoptosis. These data indicated that impaired CM proliferation during early fetal life results in a shortage of CMs at the neonatal stage in novex-3 KO mice.

### Enlargement of individual CMs, Ca^2+^ dysregulation, contractile dysfunction, chamber dilation, and poor survival in novex-3 KO mice

**Figure 3:**
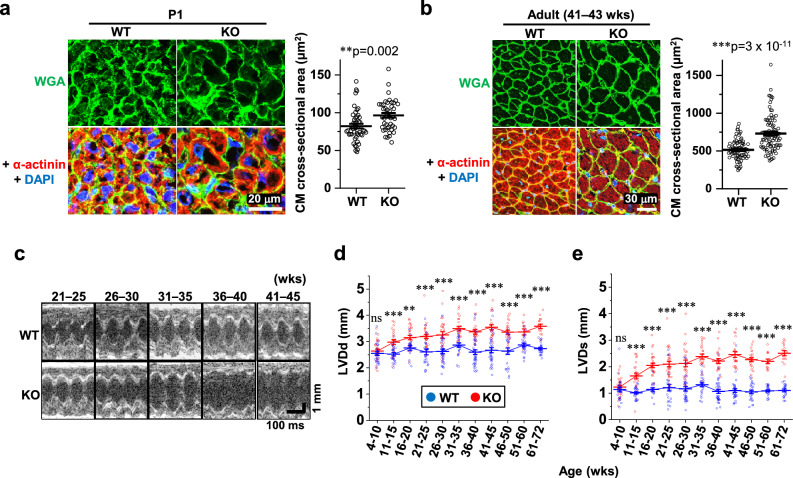

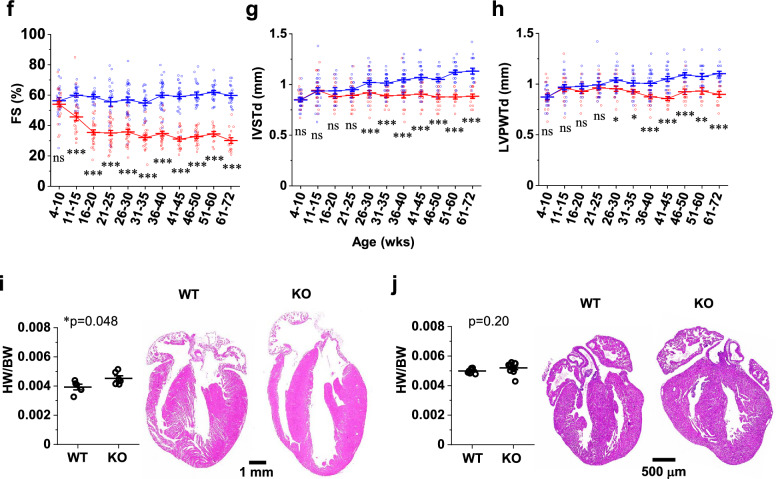
Enlargement of individual CMs, contractile dysfunction, and chamber dilation during postnatal life in novex-3 KO mice. (**a, b**) Quantification of CM cell size at P1 **(a)** and in adults (41–43 weeks) **(b)** as a cross-sectional area in the transverse direction from wheat germ agglutinin (WGA)-labeled heart sections costained with sarcomeric α-actinin (as a CM marker) and DAPI. (**a**)**:** n = 47 CMs from 6 mice for WT, n = 43 CMs from 5 mice for KO. (**b**)**:** n = 80 CMs from 8 mice for WT, n = 91 CMs from 9 mice for KO. **p < 0.01 and ***p < 0.001 as compared to WT by Student’s two-tailed unpaired t-test. Error bar = SEM. (**c**–**h**) Left ventricular internal diameter at end diastole (LVDd, **d**), left ventricular internal diameter at end systole (LVDs, **e**), interventricular septum thickness at end diastole (IVSTd, **g**), and left ventricular posterior wall thickness at end diastole (LVPWTd, **h**) were measured in sedated WT (blue) and KO (red) mice at the indicated age by two-dimensional transthoracic M-mode echocardiography. Fractional shortening (FS, **f**) was calculated as ([LVDd − LVDs]/LVDd) × 100 (%), and was used as an index of cardiac contractile function. Representative tracings are shown in (**c**). *p < 0.05, **p < 0.01, and ***p < 0.001 as compared to WT of the same age by Student’s two-tailed unpaired t-test. NS: not significant. n > 11 mice per group at each age which partially includes repetitive measurements of the same animal at different age. Error bar = SEM. (**i**) (Left) Quantification of heart-to-body weight ratio (HW/BW) in adults from WT/KO mice. n = 5–6 mice per group (41–59 weeks). *p < 0.05 as compared to WT by Student’s two-tailed unpaired t-test. Error bar = SEM. (Right) Representative H&E stainings for adult heart sections from WT (45 weeks)/KO (43 weeks) mice. **j**. (Left) Quantification of heart-to-body weight ratio (HW/BW) at P0 from WT/KO mice. n = 7–10 mice per group. Exact *p* values as compared to WT by Student’s two-tailed unpaired t-test are shown. Error bar = SEM. (Right) Representative H&E stainings for P0 heart sections from WT/KO mice.

**Figure 4:**
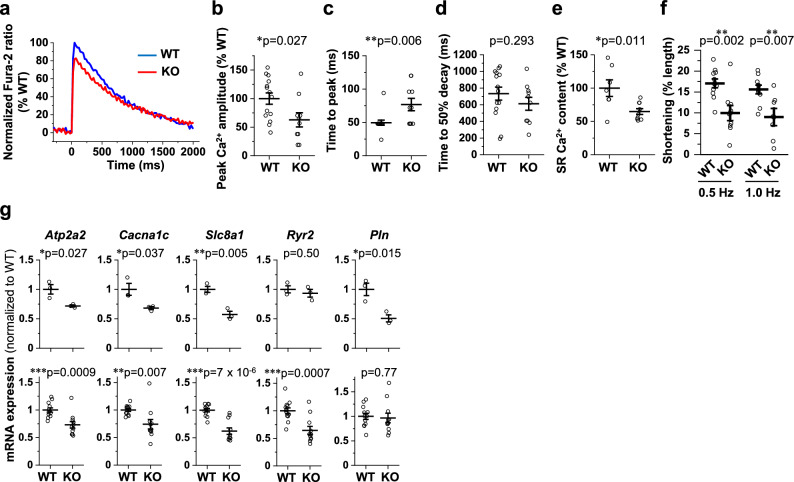

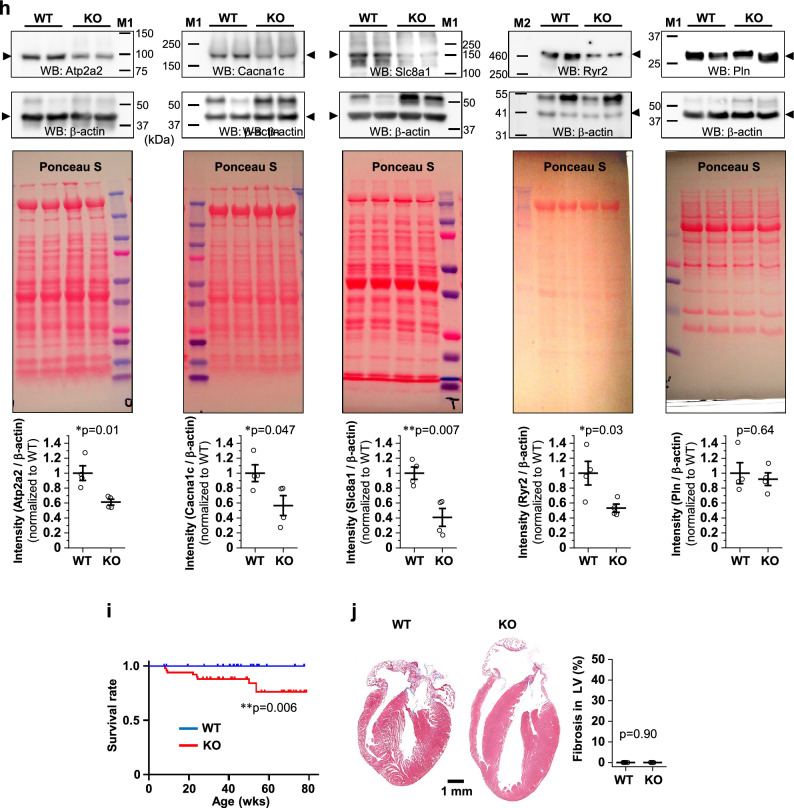
Impaired Ca^2+^ handling, contractile dysfunction, and poor survival in adult novex-3 KO mice. (**a**–**f**) Ca^2+^ transients and cell shortening were measured in dissociated CMs from adult WT/KO mice. Representative Fura-2 ratio tracings of CMs (WT: blue, KO: red) stimulated at 0.5 Hz are shown as normalized to the peak value in WT set at 100% (**a**). Quantitative analysis for peak Ca^2+^ amplitude (% normalized to WT) (**b**), time to peak (**c**), time to 50% decay (**d**), and sarcoplasmic reticulum (SR) Ca^2+^ content (% normalized to WT) (**e**) are shown. Cell shortening (% of initial cell length) stimulated at indicated frequencies are shown in (**f**). n = 7–14 CMs from 2 to 3 mice per group (36–66 weeks). In (**b**)–(**e**), *p < 0.05 and **p < 0.01 as compared to WT by Student’s two-tailed unpaired t-test. In (**f**), **p < 0.01 as compared to WT under the same stimulating frequency by Student’s two-tailed unpaired t-test. Error bar = SEM. (**g**) qPCR analysis of Ca^2+^ handling gene transcripts in P0 (top, n = 3 mice per group) and adult (bottom, n = 11–12 mice per group, 31–70 weeks) hearts from WT/KO mice. Data are shown as normalized to WT. *p < 0.05, **p < 0.01, and ***p < 0.001 as compared to WT by Student’s two-tailed unpaired t-test. Error bar = SEM. (**h**) Western blot for calcium handling genes in adult hearts (30–70 weeks) from WT/KO mice. The representative blot for two mice in each condition is shown. The blot for β-actin (marked as arrowhead) and Ponceau S staining of the membrane were done as a loading control. M1: size marker (10–250 kDa). M2: size marker (30–460 kDa). Original blots are presented in Supplementary Fig. 6. Densitometric analysis with β-actin as a reference control is shown as normalized to WT. n = 4 mice per group. *p < 0.05 and **p < 0.01 as compared to WT by Student’s two-tailed unpaired t-test. Error bar = SEM. (**i**) Overall survival curves of WT (blue) and KO (red) mice were analyzed by Kaplan–Meier method. The vertical line in each plot indicates the censored data. n = 44–51 mice per group. **p < 0.01 as compared to WT by log-rank test. (**j**) (Left) Representative Masson’s trichrome stainings of heart sections from WT (45 weeks) and KO (43 weeks) mice. (Right) Quantitative analysis of left ventricular fibrosis. n = 5–6 mice per group (37–49 weeks). Exact *p* values as compared to WT by Student’s two-tailed unpaired t-test are shown. Error bar = SEM.

In neonates (P1), we found that the CM cell size was significantly larger in KO mice than in WT mice (Fig. 3a). This was similarly observed in adults (41–43 weeks, Fig. 3b). In addition, we found increased expression of genes that are recognized as immature undifferentiated markers^4^ in KO hearts in neonates, but not in adults, suggesting impaired CM differentiation in neonates (Supplementary Fig. 2). Echocardiography revealed progressive left ventricular dilation both at diastole and systole concomitant with a profound decline in cardiac contractile function in the KO mice, as estimated by fractional shortening (Fig. 3c–f). Interventricular septum thickness (Fig. 3g) and left ventricular posterior wall thickness (Fig. 3h) were slightly but significantly decreased in KO mice. These changes were represented by a slightly increased heart-to-body weight ratio in adults (Fig. 3i), but not in neonates (Fig. 3j). The Ca^2+^ transient measurements in dissociated CMs from adult mice (Fig. 4a) demonstrated a reduction in the peak amplitude (Fig. 4b) and a delay in the time to peak (Fig. 4c), indicating poorer Ca^2+^ mobilization in KO mice than in WT mice. Although the time to 50% decay was not altered (Fig. 4d), the sarcoplasmic reticulum (SR) Ca^2+^ content was decreased (Fig. 4e), perhaps indicating impaired Ca^2+^ re-uptake into the SR in the KO mice. Cell shortening in response to electrical stimuli was decreased in KO mice at all the frequencies tested, indicating impairment of the CM contractile properties (Fig. 4f). Subsequent qPCR analysis showed depressed expression of major Ca^2+^ handling genes in KO hearts both in neonates and adults (Fig. 4g). This was confirmed at the protein level in adult hearts (Fig. 4h). Survival analysis indicated an exacerbated survival rate in KO mice (Fig. 4i). Meanwhile, almost no fibrosis was observed in adult WT/KO hearts (Fig. 4j), which might be linked to the absence of apoptosis and thus the loss of myocardium (Fig. 2i). These data suggest that the absence of novex-3 compromised fetal CM proliferation and induced compensatory enlargement of individual CMs in neonates. In adults, novex-3 deficiency resulted in chamber dilation with wall thinning and systolic dysfunction, associated with Ca^2+^ dysregulation, resulting in a reduced life span. In this study, we used mice at > 30 weeks of age (mostly 30–45 weeks) as adults, because the echocardiographic analyses demonstrated a clear KO phenotype beyond 30 weeks (Fig. 3c–h).

### Stiffer CM nuclei positioned peripherally with stabilized circumnuclear microtubules in novex-3 KO mice

**Figure 5:**
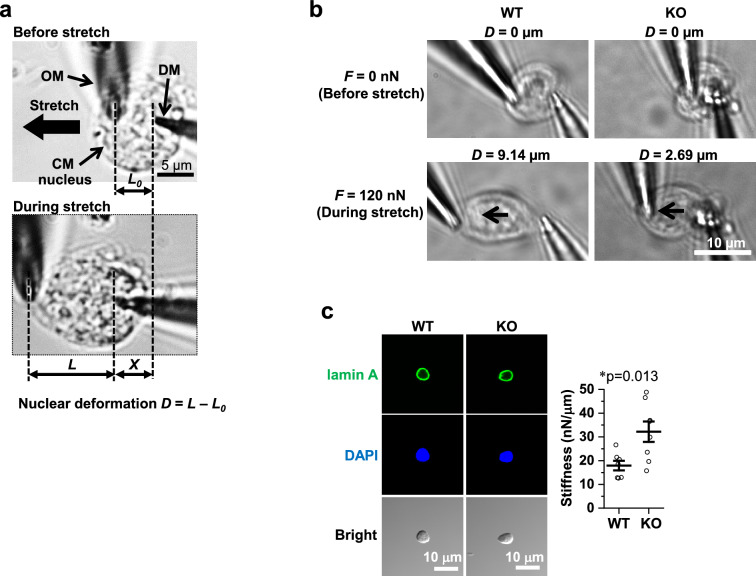

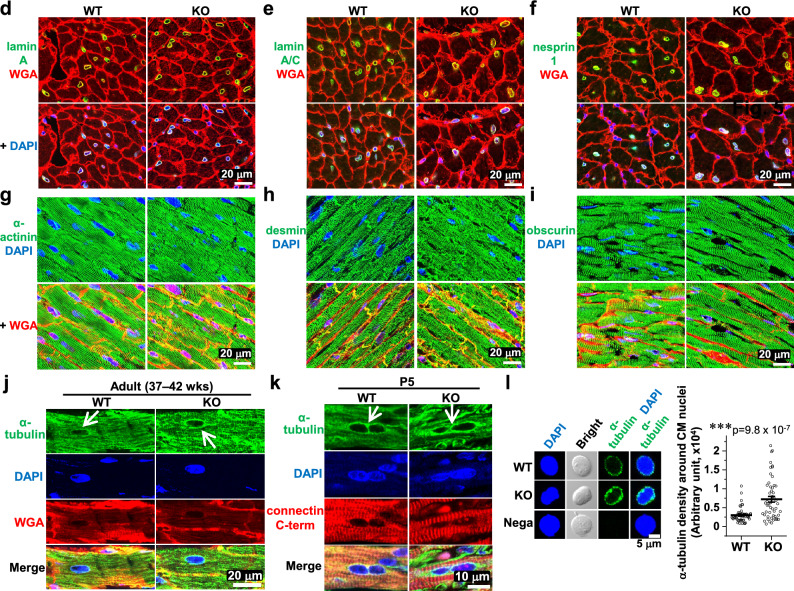

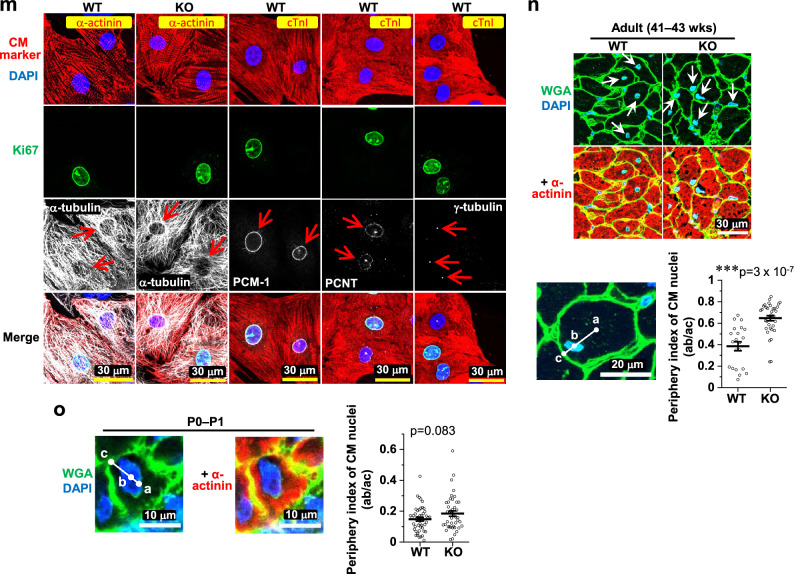
Stiffer CM nuclei positioned peripherally with stabilized circumnuclear microtubules in novex-3 KO mice. (**a,b**) Microneedle-based tensile test (adapted from^12^). One microneedle, designated as an operation microneedle (OM), was rigid and was moved with a three-axis motorized micromanipulator to stretch the nucleus. The other microneedle, designated as a deflection microneedle (DM), was flexible, to obtain the force applied to the nucleus by measuring its deflection. A single isolated CM nucleus was captured by putting the microneedle tips into the nucleus. The nucleus was then lifted off the chamber bottom and stretched horizontally at a rate of 0.5 μm/s by moving the OM along the surface of the chamber bottom. The stiffness was calculated from the slope of the force (F) versus nuclear deformation (D) curve. D was defined as L − L_0_, where L was the distance between the tips of the two microneedles along the axis of stretch (L_0_, the initial distance before stretch). X: the deflection of the DM. (**c**) (Left) Isolated P1 CM nuclei from WT/KO mice stained with lamin A and DAPI. (Right) Stiffness of P1 CM nuclei isolated from WT/KO mice. n = 7–8 nuclei per group. *p < 0.05 as compared to WT by Student’s two-tailed unpaired t-test. Error bars = SEM. (**d–i**) Immunofluorescence of lamin A (**d**), lamin A/C (**e**), nesprin1 (**f**), α-actinin (**g**), desmin (**h**), and obscurin (**i**) costained with WGA and DAPI in adult heart tissue sections from WT/KO mice (41–43 weeks). (**j,k**) Immunofluorescence of α-tubulin costained with WGA (**j**) or antibody against connectin C-term (**k**) together with DAPI in adult (37–42 weeks) (**j**) or neonatal P5 (**k**) heart tissue sections from WT/KO mice. Arrows denote the circumnuclear cage-like α-tubulin microtubule cluster that surrounds the nucleus, typically found in postnatal CMs. (**l**) (Left) Isolated P0–P2 CM nuclei from WT/KO mice stained with α-tubulin and DAPI. Nega: no primary antibody for α-tubulin. (Right) Quantification of α-tubulin density around CM nuclei. See methods for details. n = 52 nuclei per group from 3–4 independent experiments (In each experiment, CM nuclei were isolated from 4 to 11 neonatal mice.). ***p < 0.001 as compared to WT by Student’s two-tailed unpaired t-test. Error bars = SEM. (**m**) Immunofluorescence of α-tubulin and centrosomal proteins PCM-1, PCNT, and γ-tubulin costained with cell cycle marker Ki67, CM marker (α-actinin or cardiac troponin I; cTnI), and DAPI in dissociated E12–E13 CMs. Ki67-negative non-cycling CMs and Ki67-positive cycling CMs were compared in the same culture (arrows). (**n**) (Top) Immunofluorescence of α-actinin (as a CM marker) costained with wheat germ agglutinin (WGA) and DAPI in heart transverse tissue sections from adult WT/KO mice (41–43 weeks). (Bottom left) A typical image depicting cell centroid (point a), nuclear centroid (point b), and a point on the cell border intersecting the line extended from the two points (point c), determined by ImageJ 1.54b. Green: WGA, Blue: DAPI. (Bottom right) Periphery index of CM nuclei quantified as the length between a and b divided by the length between a and c. As the value approaches 0, it indicates a centrally positioned nucleus. As the value approaches 1, it indicates a peripherally positioned nucleus. n = 21 CM nuclei from 7 mice for WT, and n = 36 CM nuclei from 9 mice for KO. ***p < 0.001 as compared to WT by Student’s two-tailed unpaired t-test. Error bar = SEM. (**o**) (Left) A typical image of α-actinin (as a CM marker), WGA, and DAPI in heart transverse tissue sections from P0 to P1 mice, depicting the point a, b, and c as in (**n**). (Right) Quantification of the periphery index of CM nuclei as in (**n**). n = 47 CM nuclei from 6 mice for WT, and n = 43 CM nuclei from 5 mice for KO. Exact *p* values as compared to WT by Student’s two-tailed unpaired t-test are shown. Error bar = SEM.

We previously found that novex-3 may provide elasticity/compliance to the CM nuclei, thereby facilitating CM cell cycle progression^12^. Therefore, in the present study, we evaluated the elasticity/compliance (stiffness) of CM nuclei by performing experiments on a single isolated CM nucleus and stretching it directly with a microneedle-based tensile test system (Fig. 5a, b)^12^. This analysis revealed that CM nuclei were stiffer (less compliant) in KO P1 neonates than in their WT counterparts (Fig. 5c), providing further support for the involvement of novex-3 in the elasticity of CM nuclei. We explored the possible underlying mechanisms leading to stiffer nuclei in KO CMs by searching for nuclear and cytoskeletal proteins exhibiting gross changes in amount and/or distribution in WT versus KO CMs that could potentially affect the compliance of the nuclei. Isolated P1 CM nuclei showed no changes in lamin A or chromosomal structure (DAPI signal) (Fig. 5c). Adult CMs (41–43 weeks) also showed no alteration in nuclear proteins, such as lamin A/C and nesprin1 (Fig. 5d–f), or cytoskeletal proteins, such as α-actinin, desmin intermediate filaments, and obscurin (Fig. 5g–i). However, we found that the circumnuclear cage-like α-tubulin microtubule cluster, which typically surrounds the nucleus in postnatal CMs^22–24^, was much more stabilized in the KO mice than in the WT mice (Fig. 5j). A similar effect was also observed at neonatal stage P5 (Fig. 5k). We corroborated these findings by quantitating the α-tubulin density around isolated CM nuclei from neonates ranging in age from P0–P2 (Fig. 5l). We next assessed whether these circumnuclear microtubules were correlated with CM cell cycle. Co-immunofluorescence stainings for α-tubulin and Ki67 in dissociated E12–E13 CMs demonstrated the presence of intense circumnuclear microtubules in Ki67-negative non-cycling CMs, but not in Ki67-positive cycling CMs, regardless of the genotype (Fig. 5m), suggesting the possible link between the circumnuclear microtubules and cell cycle activities. These differential effects between Ki67-positive and -negative CMs were not observed for the centrosomal proteins PCM-1, PCNT, and γ-tubulin (Fig. 5m), which in our experimental settings showed typical intracellular distributions during mitosis (Supplementary Fig. 3). In KO CMs, we also observed abnormal nuclear positioning adjacent to the cell periphery in transverse sections, weakly in neonates and intensely in adults (Fig. 5n, o). Collectively, the data supported the idea that the absence of novex-3 provoked abnormal nuclear mechanics, including stiffer nuclei positioned peripherally with stabilized circumnuclear microtubules, which might be associated with impaired CM proliferation.

## Discussion

More than two decades have passed since the discovery of novex-3^16^, and yet its physiological functions remain poorly understood. Our recent in vitro analyses demonstrated that novex-3 is expressed in CM nuclei exclusively during fetal life, where it provides elasticity/compliance to CM nuclei and promotes CM proliferation in the fetus^12^, suggesting a non-sarcomeric function. In the present study of novex-3 KO mice, we confirmed that these phenotypes are recapitulated in vivo. In the absence of novex-3, CM proliferation was compromised in the fetus (Fig. 2), and a reduced number of enlarged CM was observed in neonates (Figs. 2, 3). In adults, novex-3 deficiency resulted in chamber dilation with wall thinning and systolic dysfunction, associated with Ca^2+^ dysregulation, resulting in a reduced life span (Figs. 3, 4). Our findings underscore the critical importance of novex-3 as an early contributor to CM proliferation in the fetus.

The precise mechanisms by which novex-3 promotes CM proliferation are currently unclear. However, the manifestation of stiffer nuclei with stabilized circumnuclear microtubules in KO CMs (Fig. 5) suggests a number of possible mechanism(s). The microtubule organizing center (MTOC), which plays a key role in microtubule nucleation, is known to relocate from the centrosome to circumnuclear cage-like arrangement in CMs around birth and this has been proposed to lead to cell cycle arrest in CMs^22,23,25^. Relocation of the MTOC from the centrosome, which is required for cell cycle progression^26^, to non-centrosomal loci upon differentiation has been described for other cell types, including neurons, skeletal muscle cells, osteoclasts, and epithelial cells^27^. In postnatal CMs, which experience the increasing workload of the heart, circumnuclear microtubules could protect the nuclei from compression forces, thereby maintaining genome organization^22,23,28,29^ and nuclear homeostasis^24^. Hence, this MTOC relocation is considered to represent the switch from the hyperplastic to the hypertrophic state in CMs that occurs around birth. The intense circumnuclear microtubules observed in Ki67-negative non-cycling CMs but not in Ki67-positive cycling CMs in fetuses is consistent with this concept (Fig. 5m).

In this context, the excessive accumulation of circumnuclear microtubules in KO CMs (Fig. 5) led us to speculate that the expression of nuclear novex-3 in fetal CMs might inhibit MTOC relocation until the optimal timing (i.e., at birth), thereby maintaining proliferative potential in fetal CMs. The disappearance of nuclear novex-3 after birth (Fig. 1) would then release this inhibitory effect and lead to MTOC relocation and cell cycle arrest. In KO CMs, the premature MTOC relocation during fetal life in the absence of novex-3 could lead to proliferation defects (Fig. 2) and the aberrant accumulation of circumnuclear microtubules (Fig. 5). We also observed abnormal nuclear positioning adjacent to the cell periphery in KO CMs, weakly in neonates and intensely in adults (Fig. 5n, o). This might also result from premature MTOC relocation, because the circumnuclear microtubules generated at the correct time would establish correct nuclear positioning^30^. To better understand the mechanisms by which novex-3 influences MTOC relocation, we performed preliminary immunofluorescence stainings using CM nuclei isolated from E17–E18 mice to investigate the subnuclear localization of novex-3 and its possible colocalization with nuclear and centrosomal proteins, most of which had previously been linked to MTOC relocation^27,30^. We found that novex-3 showed a punctate distribution throughout the nuclear envelope, and these punctate foci were sometimes aggregated into a bright structure that was located in the vicinity of nuclear envelope proteins lamin A/C, sun2, and nesprin1 (Supplementary Fig. 4). Notably, this bright structure unequivocally and consistently colocalized with foci of all four centrosomal proteins analyzed (γ-tubulin, PCNT, PCM-1, and AKAP9). This colocalization was seen in about 60% of the analyzed nuclei (61%, n = 36 nuclei for γ-tubulin; 57%, n = 47 nuclei for PCNT; and 60%, n = 40 nuclei for AKAP9). This consistent coexistence of novex-3 with centrosomal proteins led us to speculate that novex-3 inhibited MTOC relocation by sequestering centrosomal proteins and thus interrupted their recruitment to the nuclear envelope in fetal CMs, rather than directly inhibiting the assembly of circumnuclear microtubules. While the significance of 60% positivity for the colocalization with centrosomal proteins is currently unclear, it may relate to cell cycle activities in individual CMs. The physical interactions between novex-3 and centrosomal proteins, and their functional consequences must be experimentally corroborated in future research.

We found that the CM nuclei were stiffer (less compliant) in KO neonates than in their WT counterparts (Fig. 5), consistent with the previous suggestion^12^. Generally, two elements, the lamin-based nuclear envelope and the DNA/histone-based chromatin structure, are thought to be responsible for nuclear stiffness^31^. Immature cells, such as stem cells, have highly dynamic, decondensed chromatin with low levels of lamin expression, resulting in compliant nuclei that enable active transcription for diverse cellular activities, such as proliferation and migration. Upon differentiation, the cells become characterized by increasing expression of lamin and chromatin condensation with an overall gene silencing, while preserving lineage-specific gene expression, resulting in stiffer nuclei that enable cell type-specific activities, such as contraction in muscle cells^32–36^. Accordingly, several researchers have proposed that cells with compliant nuclei tend to undergo active proliferation^37–39^ and migration^40,41^, although the underlying mechanism is not entirely clear. The novex-3 KO CMs with stiffer nuclei showed impaired proliferation in the present study (Figs. 2, 5), in agreement with this notion. However, although not extensively analyzed, the lamins and chromosomal structure did not appear to undergo any gross changes in KO versus WT CM nuclei, whereas the KO CMs showed significant accumulation of circumnuclear microtubules (Fig. 5). This suggests that, in addition to lamins and chromatins, circumnuclear microtubules may also contribute to nuclear stiffness and could be responsible for regulating proliferation in CMs.

Collectively, we suggest that nuclear novex-3 facilitates fetal CM proliferation by maintaining the functional centrosome intact to inhibit MTOC relocation until birth, thereby achieving a compliant nucleus that facilitates the dynamic transcription program required for cell division. Clearly, future research must test this possibility.

While we focused on the nuclear novex-3, an important unanswered question is how sarcomeric novex-3 is implicated in KO mice. The initial study showed that novex-3 forms an elastic Z-disc–to–I-band linking system with obscurin^16^, although this role could not be confirmed in a later study^21^. In the present study, no differences were noted for the distributions of α-actinin, desmin, or obscurin in KO CMs at the level of immunofluorescence analysis (Fig. 5), suggesting that the sarcomere organization was not grossly affected. However, more in-depth studies including ultrastructural analysis are necessary to elucidate how the absence of novex-3 affects sarcomere organization. It is possible that the absence of sarcomeric novex-3 aggravated the contraction–relaxation cycle of the sarcomere, leading to Ca^2+^ dysregulation and contractile dysfunction in KO mice (Figs. 3, 4).

In summary, this work provides important new insights into our understanding of the pathophysiological role of novex-3, an ill-defined short variant of connectin (titin). Novex-3 clearly has a non-sarcomeric function and plays a pivotal role as an early contributor to CM proliferation in mice. Although the mechanisms are not entirely clear, the presence of compliant nuclei with intact centrosomes without circumnuclear microtubules appears to be the key driver of novex-3 promotion of CM proliferation. Future research should therefore focus on identifying the precise mechanisms of this function of novex-3 in relation to MTOC relocation and nuclear mechanics, as well as dissecting nuclear versus sarcomeric shuttling of novex-3.

### Limitations of the study

In this study, four limitations should be considered. One was that we could not reach definitive conclusions as to how novex-3 promotes CM proliferation. Further research is obviously necessary to validate the possible mechanisms described in the Discussion. Especially important is the functional correlation of nuclear novex-3 with perinatal MTOC relocation and associated nuclear mechanics, which are beyond the scope of this paper but clearly deserve additional study. A second limitation was that although novex-3 is a striated muscle-specific protein, skeletal muscle phenotypes were not considered, except for the KO validation (Fig. 1). The reasons for this are multifactorial: (1) In line with our previous report^12^, the main purpose of the present study was to reveal the function of novex-3 in cardiac pathophysiology, especially in relation to CM proliferation; (2) A previous study suggested that novex-3 transcripts are less abundant in skeletal muscle than in myocardium^16^; (3) Overt phenotypes, such as muscular weakness and gait disorder, were not apparent by visual inspection in KO mice, in contrast to the severe phenotypes observed in CMs. However, any abnormality arising from skeletal muscles might contribute to the phenotypes seen in novex-3 KO mice. Therefore, this limitation needs to be carefully considered when interpreting the current data. A third limitation was that the contribution of nuclear versus sarcomeric novex-3 could not be clearly distinguished. One possible approach would be to introduce a loss-of-function mutation in the obscurin binding site of novex-3, which is located in a novex-3-specific region^16^, to disrupt the putative novex-3-obscurin interaction, thereby specifically inactivating the function of sarcomeric novex-3. A fourth limitation was a small sample size in some experiments (Figs. 2a, b, d and 4g, h), which limited our ability to draw definitive conclusions. Therefore, the scientific significance of the findings should be interpreted carefully.

## Methods

### Generation of novex-3 KO mice using CRISPR/Cas9

Mice with a C57BL/6N background were housed in a temperature-controlled room under a 12-h light:12-h dark cycle conditions and were fed a standard chow diet and water ad libitum. Novex-3 KO mice were generated as follows: Guide RNAs targeting the N-termini of novex-3-specific exon (ENSMUST00000099980.10, Fig. 1a) were designed with the online design tool (Benchling at https://benchling.com/signin/welcome#, and CRISPOR at http://crispor.tefor.net/). The resulting two candidate guide RNAs (#1: 5′-TCTCGGTTCTTACGACCCGACGG-3′, #2: 5′-CGGCTTGAAATATTCAGGCCAGG-3′, PAM sequence underlined) were assessed for in vitro cleavage of the target DNA sequence using Guide-it sgRNA In Vitro Transcription and Screening System (Takara Bio, Japan), which favored the guide RNA #1 as the final candidate (Supplementary Fig. 5). The guide RNA #1 was synthesized as a complex of CRISPR RNA (crRNA) and trans-activating crRNA (tracrRNA) (Integrated DNA Technologies, USA), and used for mouse genome editing. Pronuclear stage mouse zygotes generated by in vitro fertilization were electroporated with the guide RNA and Cas9 nuclease (Integrated DNA Technologies) using super electroporator NEPA21 (NEPA Gene, Japan), and about 30 embryos at the two-cell stage were transferred into the oviduct of pseudo-pregnant recipient mouse on an ICR background^42^. Genotyping of founder animals by Sanger sequencing and TIDE tool (https://tide.nki.nl/) resulted in a line with a 5 bp deletion in the target region causing a frameshift that was predicted to create a novel stop codon (Fig. 1). Subsequent mating with WT animals and germ line transmission established this line as a novex-3 deficient mice (Fig. 1). No mutations were detected for the top 3 genes (Hoxa7, Plekha5, and Fank1) predicted to be most susceptible to off-target effects. This study was performed in accordance with the recommendations of the ARRIVE guidelines and Institutional Animal Care and Use Committee at the Kawasaki Medical School. All of the animals were handled according to approved institutional protocols of the Kawasaki Medical School, and every effort was made to minimize suffering. All experiments were performed in accordance with the relevant guidelines and regulations of the Kawasaki Medical School. All experimental protocols were approved by a named institutional committee (Institutional Animal Care and Use Committee at the Kawasaki Medical School).

### Immunofluorescence

Frozen heart tissues embedded in OCT compound (Tissue-Tek; Sakura, UAE) were cut into 8 µm sections with a cryostat (Leica, Germany), permeabilized, blocked with Blocking-One (Nacalai Tesque, Japan), and labelled with primary antibodies, followed by fluorochrome-conjugated secondary antibodies. Counterstaining for DAPI (chromosomal DNA) and wheat germ agglutinin (WGA; cell membrane) was also performed. Sections were covered with a fluorescence mounting medium (Dako, USA) and imaged using a confocal scanning system mounted on a IX81 inverted microscope (FV-1000, Olympus, Japan) with a 60× oil-immersion objective lens (UPlanSApo, Olympus), or an inverted fluorescence microscope (BZ-X710, Keyence, Japan) with a 40× objective lens (PlanApoλ, Nikon, Japan). Essentially the same staining protocol was applied for dissociated CM cultures, with the addition of fixation step by methanol, methanol/acetone, or 4% paraformaldehyde before permeabilization. For immunostaining of isolated nuclei, the CM nuclei were isolated from dissociated CM culture using a Nuclei EZ Prep Nuclei Isolation Kit (Sigma-Aldrich, USA) as previously described ^12^. Samples containing nuclei were fixed with methanol, methanol/acetone, or 4% paraformaldehyde, blocked, and labelled with primary antibodies, followed by fluorochrome-conjugated secondary antibodies. For α-tubulin staining of tissue samples, the following protocol was applied (adapted from^43^). Tissues were collected from mice, fixed in 4% paraformaldehyde, embedded in paraffin, and sectioned at a thickness of 5 μm. Sections were deparaffinized, permeabilized with 1% Tween-20, and immersed in 100 °C target retrieval solution, pH 9.0 (S2367, DAKO) on a microwave pressure cooker for 10 min. After cooling to room temperature, sections were blocked with Blocking-One and labelled with a primary antibody for α-tubulin (3873, Cell Signaling, USA) for 3–4 nights at room temperature, followed by fluorochrome-conjugated secondary antibodies. The primary antibodies used were for Ki67 (ab16667, Abcam, UK), Ki67 (ab279653, Abcam), phospho-histone H3 at Ser-10 (06-570, Merck Millipore, USA), α-tubulin (ab6160, Abcam), α-tubulin (3873, Cell Signaling), γ-tubulin (sc51715, Santa Cruz, USA), PCM-1 (HPA023370, Sigma-Aldrich), PCM-1 (sc398365, Santa Cruz), PCNT (ab220784, Abcam), PCNT (611815, BD Transduction, USA), AKAP9 (611518, BD Transduction), sarcomeric α-actinin (A7811, Sigma-Aldrich), desmin (D1033, Sigma-Aldrich), cardiac troponin I (Tnni, ab56357, Abcam), nesprin1 (MABT843, Merck Millipore), lamin A (ab26300, Abcam), lamin A/C (ab8984, Abcam), sun2 (MABT880, Merck Millipore), obscurin (HPA021186, Sigma-Aldrich), connectin C-terminal region (TTN-9, Myomedix, Germany), novex-3 (TTN-2, Myomedix, designated as ab1 in Fig. 1a)^16^, and novex-3 (raised against a synthetic peptide EKDVKEFEKQVKIV [AA 5222–5235] of mouse novex-3, Eurofins Genomics, Germany, designated as ab2 in Fig. 1a). Detailed information of the antibodies is summarized in Supplementary Table 1. When using mouse-derived antibodies, the Mouse on Mouse (M.O.M.) Basic Kit (Vector, USA) was used.

### Western blotting and SDS-vertical agarose gel electrophoresis

Tissues were collected from mice and snap frozen in liquid nitrogen, minced and homogenized using a Kinematica Polytron homogenizer (PT1600E/2500E; Fisher Scientific, USA) in urea-containing buffer (8 M urea, 2 M thiourea, 3% SDS, 75 mM DTT, 0.03% bromophenol blue and 0.05 M Tris–HCl pH 6.8) to obtain whole protein extracts. Protein samples were heated at 60–65 °C for 10 min and separated by SDS-PAGE on a hand-cast gel (3–10%, 3–12%, 4–12%, or 15%) or a precast gel (4–15%, 7.5%, or 10%) (Mini-PROTEAN TGX; Bio-Rad, USA) at 5–30 mA for 1–2 h in the electrophoresis buffer (25–50 mM Tris, 192–384 mM glycine and 0.1–0.2% SDS supplemented with or without 10 mM β-mercaptoethanol). A standard for high molecular weight proteins (up to 460 kDa) was included (HiMark Pre-Stained Protein Standard, Thermo-Fisher, USA). The samples were then transferred onto nitrocellulose (Bio-Rad) or PVDF (GE Healthcare, USA) membranes using HorizeBLOT 2 M (WSE-4025, ATTO Corporation, Japan) at 20–25 V constant for 10–45 min, Trans-Blot Turbo (Bio-Rad) at 2.5 A constant for 3 min, or in the transfer buffer (25 mM Tris, 192 mM glycine and 20% methanol supplemented with or without 10 mM β-mercaptoethanol or 1% SDS) at 0.07–0.15 A constant for overnight. The membranes were blocked with 5% nonfat milk in TBS/T, probed with primary antibodies for novex-3 (TTN-2, Myomedix, designated as ab1 in Fig. 1a)^16^, connectin N-terminus (raised in our lab, designated as ab3 in Fig. 1a)^44^, Aurkb (A5102, Sigma-Aldrich), Nusap1 (12024-1-AP, Proteintech), Ccna2 (ZRB1590, Sigma-Aldrich), Atp2a2 (MA3-919, Thermo-Fisher), Cacna1c (ACC-003, Alomone), Slc8a1 (raised in our lab)^45^, Ryr2 (MA3-916, Thermo-Fisher), Pln (8495, Cell Signaling), and β-actin (sc81178, Santa Cruz), followed by secondary horseradish peroxidase (HRP)-conjugated IgG (Agilent Technologies, USA or GE Healthcare) and finally visualized by enhanced chemiluminescence (Western Lightning ECL-Pro; PerkinElmer, USA) using Amersham Imager 680 or LAS4000mini luminescent image analyser (GE Healthcare), as previously described^46^. Ponceau S staining of the membrane was done as a loading control. The intensity of each band was quantified using an Image J Macro, Band/Peak Quantification Tool^47^ (National Institutes of Health). For protein fractionation analysis, a subcellular protein fractionation kit (Thermo-Fisher) was used to separate the sample into cytoplasmic, membrane, and nuclear fractions. Each sample was mixed with Pierce Lane Marker Non-Reducing Sample Buffer (39001, Thermo-Fisher) containing 50 mM DTT or with the aforementioned urea-containing buffer, and heated at 60–65 °C for 10 min or 100 °C for 5 min. Successful fractionation was validated by immunoblotting for β-tubulin (cytoplasmic), serca2 (membrane), and fibrillarin (nuclear) (Supplementary Fig. 1f.). Antibodies used were for β-tubulin (2128, Cell Signaling), serca2 (Atp2a2, MA3-919, Thermo-Fisher), and fibrillarin (2639, Cell Signaling). For SDS-vertical agarose gel electrophoresis, the protein samples were electrophoresed on 1% vertical agarose gels (30% glycerol, 50 mM Tris, 384 mM glycine, and 0.1% SDS) at 10 mA for ~ 2 h^48^. The proteins were visualized with Coomassie brilliant blue (Bio-Safe CBB G-250, Bio-Rad), and imaged by Odyssey CLx Imager (LI-COR Biosciences, USA). The intensity of each band was quantified using ImageJ 1.54b (National Institutes of Health). All extraction procedures included a protease inhibitor cocktail (Thermo-Fisher). Detailed information of the antibodies is summarized in Supplementary Table 1.

### TdT-mediated dUTP Nick End Labeling (TUNEL) assay

Frozen heart tissues embedded in OCT compound (Tissue-Tek; Sakura) were cut into 8 µm sections with a cryostat (Leica), dried, and fixed in 4% paraformaldehyde. Terminal deoxynucleotidyl transferase (TdT)-mediated labeling reaction was performed using TdT In Situ Apoptosis Detection Kit—Fluorescein (4812-30-K, R&D systems, USA) with some modifications. Permeabilization steps were carried out by 0.25% Triton X-100. TdT enzyme was purchased from Takara Bio, and used at 0.3 U/μL. After the TdT reaction, samples were blocked with Blocking-One (Nacalai Tesque), and immunostained with primary antibodies for PCM-1 (as a CM marker, HPA023370, Sigma-Aldrich), followed by fluorochrome-conjugated secondary antibodies, together with DAPI, WGA (to delineate cell border), and Streptavidin-FITC (to detect biotinylated nucleotides in fragmented DNA). Sections were covered with a fluorescence mounting medium (Dako) and imaged using an inverted fluorescence microscope (BZ-X710, Keyence) with a 40 × objective lens (PlanApoλ, Nikon). Positive controls were treated with 100 U/mL DNase I (Thermo-Fisher) before the TdT reaction to generate DNA breaks. Negative controls were processed with TdT enzyme omitted from the labeling reaction. TUNEL-positive CMs were quantified from the total CMs that were identified by PCM-1 and DAPI signals using ImageJ 1.54b (National Institutes of Health).

### Quantitative PCR (qPCR)

Tissues were collected from mice, cut into small pieces, and immediately immersed in RNAlater Stabilization Reagent (Qiagen, Germany). The stabilized tissues were homogenized with a Kinematica Polytron homogenizer (PT1600E/2500E; Fisher Scientific), and total RNA was isolated using the ISOGEN or ISOGEN-II systems (Nippon Gene, Japan). After assessing RNA yield and quality using a NanoDrop One spectrophotometer (Thermo-Fisher), the RNA samples were reverse-transcribed with PrimeScrip RT Master Mix (TaKaRa Bio), and quantitative real-time PCR was performed using TaqMan Fast Advanced Master Mix in a StepOnePlus real-time PCR system (Applied Biosystems, USA). Quantification of each mRNA was carried out with *Actb*, *Ubc*, or *18S rRNA* as reference genes, using the ΔΔC_T_ method^4^. Taqman probe for novex-3 (ENSMUST00000099980.10) was custom designed at the boundary between exon 44 and exon 45 (novex-3-specific exon) not to affect the cleavage site of the guide RNA using Custom TaqMan Assay Design Tool (https://www.thermofisher.com/order/custom-genomic-products/tools/gene-expression/). Taqman probe Mm00658612_g1 (Thermo-Fisher) targeting the common C-terminal region of full-length connectin was used for detecting major connectin isoforms, i.e., N2BA and N2B in cardiac muscles and N2A in skeletal muscles (Fig. 1a).

### Quantification of α-tubulin density around isolated CM nuclei

The CM nuclei were isolated from dissociated CM culture obtained from P0–P2 mice using a Nuclei EZ Prep Nuclei Isolation Kit (Sigma-Aldrich) as previously described^12^. The integrity of the isolated nuclei was verified by staining patterns of lamin A and DAPI, and bright field image (Fig. 5c). Density of α-tubulin around isolated CM nuclei was quantified as follows using ImageJ 1.54b (National Institutes of Health). The region of interest (ROI) for a single nucleus was first determined by applying wand tool on the binary image generated by maximum projection of the DAPI signal through Z-stacks. α-tubulin density around CM nuclei was then quantified as a mean gray value of the α-tubulin signal summed through Z-stacks within the nuclear ROI. Use of the mean gray value excluded the confounding influence by nuclear size.

### Microneedle-based tensile test of a single isolated *CM nucleus*

The CM nuclei were isolated as mentioned above. Microneedle-based tensile test was performed as previously described^12^. The experimental set up was slightly modified from a laboratory-made tensile test system consisting of an inverted microscope (IX-73; Olympus), a 60× oil immersion objective lens (PlanApo N 60×; Olympus), a digital CMOS camera (ORCA flash 4.0; Hamamatsu Photonics, Japan) and a pair of glass microneedles each connected to three-axis motorized micromanipulators (EMM2; Narishige, Japan). One microneedle, designated as an operation microneedle, was rigid to stretch the nucleus. The other microneedle, designated as a deflection microneedle, was flexible enough to deflect as the nucleus was stretched. The spring constant of the microneedle was determined by a cross-calibration method^49^. A single isolated nucleus was captured by piercing the tips of two microneedles into the periphery of the nucleus (Fig. 5a, b). After the nucleus was lifted off the chamber bottom by moving the two microneedles upward, the nucleus was stretched horizontally at a rate of 0.5 μm/s by moving the operation microneedle along the surface of the chamber bottom. The experiments were performed at room temperature. The positions of the tips of microneedles were measured using MetaMorph Offline software (version 7.7.0.0; Molecular Devices, USA). The nuclear deformation, *D*, was defined as *L* − *L*_0_, where the *L* and *L*_0_ were the distances between the tips of the two microneedles along the stretch axis during and before stretch, respectively. The force, *F*, applied to the nucleus was calculated by multiplying the deflection of the deflection microneedle, *X*, by its spring constant. The stiffness was defined as the slope of the force (*F*) versus nuclear deformation (*D*) curve within the deformation range of 0–10 μm, based on the assumption that the curve is piecewise linear.

### Histology

Heart tissues were collected from mice, fixed in 4% paraformaldehyde, embedded in paraffin, and longitudinal sections were cut at a thickness of 3 μm. The sections were stained with Hematoxylin–eosin (H&E) or Masson’s trichrome according to standard procedures, and observed with a light microscope (BZ-X710, Keyence) with a 10 × objective lens (PlanFluor, Nikon). Fibrosis was quantified from Masson’s trichrome stainings using ImageJ 1.54b (National Institutes of Health) based on the scar area (blue) and healthy area (red) in the left ventricle.

### Echocardiography

Two-dimensional transthoracic echocardiography was performed to evaluate cardiac function using an Aplio 300 system with a 14-MHz transducer (Toshiba Medical System, Japan)^4^. M-mode tracings were used to measure the left ventricular internal diameter at end diastole (LVDd), left ventricular internal diameter at end systole (LVDs), interventricular septum thickness at end diastole (IVSTd), and left ventricular posterior wall thickness at end diastole (LVPWTd). Fractional shortening (FS) was calculated as ([LVDd − LVDs]/LVDd) × 100 (%), and was used as an index of cardiac contractile function. All examinations were performed on conscious mice to prevent anaesthesia-related impairment of cardiac function. In these non-sedated mice, an FS < 65% was considered indicative of the impaired cardiac function^50^.

### RNA-seq

Heart tissues from P1 WT/KO mice were collected, cut into small pieces, and immediately immersed in RNAlater Stabilization Reagent (Qiagen) (n = 2 mice per group and each sample contained 2 to 3 P1 hearts to obtain sufficient amount of RNA.). The stabilized tissues were homogenized with a Kinematica Polytron homogenizer (PT1600E/2500E; Fisher Scientific), and total RNA was isolated using ISOGEN or ISOGEN-II system (Nippon Gene). After assessing RNA yield and quality using a 2100 Bioanalyzer (Agilent Technologies), RNA-seq libraries were generated using the NEBNext Ultra II Directional RNA Library Prep Kit (New England Biolabs, USA). The quality of the libraries was checked using the 2200 TapeStation (Agilent Technologies). Paired-end sequencing of the libraries was performed on an Illumina NovaSeq 6000 platform (Hokkaido System Science, Japan). The obtained data were processed as follows: known adapters and low-quality regions of the reads were trimmed using cutadapt 1.1 (https://cutadapt.readthedocs.io/en/stable/) and Trimmomatic 0.32 (http://www.usadellab.org/cms/index.php?page=trimmomatic), respectively. The reads were mapped to the mouse reference genome (Mus musculus GRCm38, Release 100) using Tophat 2.0.14 (http://ccb.jhu.edu/software/tophat/index.shtml). Gene expression between samples was compared by calculating normalized expression values for each transcript as fragments per kilobase of exon model per million mapped fragments (FPKM) using Cufflinks 2.2.1 (http://cole-trapnell-lab.github.io/cufflinks/). Based on the threshold ratios of > 1.5 (up) and < 0.67 (down), 811 and 812 genes, respectively, were identified as up- and down-regulated, which was summarized in Supplementary Data 1. Based on the expression data, statistical enrichment test for reactome pathways, biological processes, and cellular components was performed using Panther (http://www.pantherdb.org/)^51^. In this test, a reference distribution was first generated using all values from the input data, which provided the basis for subsequent evaluation of the specific gene category. RNA-seq data have been deposited in DDBJ sequencing read archive (DRA) under the accession number DRA016576 (https://ddbj.nig.ac.jp/search).

### Primary CM dissociation from adult mice

Primary CMs were dissociated from the ventricles of adult mice as previously described^45^. Briefly, the heart was excised from euthanized mice and a cannula was inserted into the aorta. Langendorff-perfusion was initiated with cell-isolation buffer (CIB; 130 mM NaCl, 5.4 mM KCl, 0.5 mM MgCl_2_, 0.33 mM NaH_2_PO_4_, 22 mM glucose, 50 μU/ml bovine insulin, 25 mM HEPES–NaOH (pH 7.4)) containing 0.4 mM EGTA. The perfusate was changed to the enzyme solution (CIB containing 1 mg/ml collagenase type II (Worthington Biochemical, USA), 0.06 mg/ml protease (Sigma-Aldrich), 0.06 mg/ml trypsin (Sigma-Aldrich) and 0.3 mM CaCl_2_). The left ventricles were cut into small pieces and further digested in the enzyme solution for 10–15 min at 37 °C by gentle agitation. In this enzyme solution, the CaCl_2_ level was increased to 0.7 mM, and 2 mg/ml BSA was supplemented. After centrifugation at 14 × g for 5 min, the pellet was resuspended in CIB containing 1.2 mM CaCl_2_ and 2 mg/ml BSA, and then incubated for 10 min at 37 °C. After centrifugation again, the cells were resuspended in Tyrode’s solution (140 mM NaCl, 5.4 mM KCl, 1.8 mM CaCl_2_, 0.5 mM MgCl_2_, 0.33 mM NaH_2_PO_4_, 11 mM glucose, 5 mM HEPES–NaOH (pH 7.4)) containing 2 mg/ml BSA.

### Cell shortening and Ca^2+^ transient measurements

Electrically induced cell shortening and Ca^2+^ transients were measured as previously described^52^. The experimental setup comprised an inverted microscope (IX-73; Olympus), a 20 × objective lens (UCplanFLN; Olympus), a CMOS camera (ORCA flash 4.0; Hamamatsu Photonics), an LED illuminator (pE-340fura; CoolLED), and an electrical stimulator system^52,53^. The Ca^2+^ transients were measured by loading dissociated CMs with 5 μM Fura-2 AM (Dojindo, Japan) for 30 min. The Fura-2-loaded cells were alternately excited at 340 and 380 nm using an LED illuminator. The Ca^2+^ content of the sarcoplasmic reticulum (SR) was evaluated by rapidly applying 10 mM caffeine and measuring the resulting Ca^2+^ transients in dissociated CMs. Data were analyzed using MetaMorph software (version 7.8.0.0; Molecular Devices).

### Primary CM dissociation from fetal/neonatal mice

Primary CMs were dissociated from fetal (E12–E17) and neonatal (P0–P3) mice as previously described^4^. For fetal CMs, pregnant mice were euthanized with Sevofrane, and fetal hearts were rapidly excised, cut into small pieces, and digested 3 to 4 times with 0.06% trypsin in PBS for 10 min at 37°C. For neonatal CMs, neonatal heart dissociation kit (130-098-373, Miltenyi Biotec, Germany) with gentleMACS Octo Dissociator with Heaters (Miltenyi Biotec) was used as per the manufacture’s protocol. After a 20 min culture to exclude non-CMs, CMs were plated onto fibronectin-coated culture vessels in DMEM-F12 supplemented with 2% FBS, 2 mM l-alanyl-l-glutamine (Nacalai Tesque), 0.2% bovine serum albumin, 0.5 mM Na-pyruvate, 0.1 mM l-ascorbic acid, 0.5% Insulin-Transferrin-Selenium (Thermo-Fisher), and penicillin/streptomycin (adapted from^27^), and cultured under standard conditions at 37 °C with 5% CO_2_. In each dissociation procedure, 5–10 fetal/neonatal hearts were pooled and used for the dissociation.

### Fixation digestion for counting total ventricular CMs

The total number of CMs in the ventricle from P13–P14 mice was evaluated with a fixation digestion method^4^. The heart was excised from mice and washed with cardioplegia solution containing 25 µM KCl. Ventricles were cut into 0.5–1 mm tissue blocks, fixed with 4% paraformaldehyde for 100 min, washed 3 times with PBS, and digested with the enzyme solution containing 3.6 mg/mL collagenase B (11-088-807-001, Sigma-Aldrich) and 4.8 mg/mL collagenase D (11-088-858-001, Sigma-Aldrich) for 24 h at 37°C by gentle agitation. The digested cells were collected, and the rod-shaped CMs were counted with a hemocytometer. The undigested tissues were digested with a new enzyme solution for an additional 24 h. These procedures were repeated until all tissues were digested.

### Baculovirus-mediated protein expression

A baculovirus-mediated protein expression system in dissociated fetal or neonatal CMs was previously established^4,46^. An intense expression of target protein in CMs was confirmed by Western blotting and fluorescence imaging of GFP, which was fused to the target sequence. In this study, a baculovirus expressing full length mouse novex-3 (XM_036160543.1) was used. Baculovirus was produced in Sf9 cells, as per the manufacturer’s instructions (Thermo-Fisher). For transduction to CMs, virus was added to the cells in DMEM without serum. After 7 h, the cells were treated with BacMam enhancer (Invitrogen, USA) for an additional 2 h, according to the manufacturer’s protocol, to increase the transduction efficiency. The medium was then replaced with DMEM containing 10% FBS.

### Quantification and statistical analysis

All data were expressed as mean plus or minus standard error of the mean (SEM). For comparisons between two groups, Student’s two-tailed unpaired t-test was performed using Microsoft Excel 2019 MSO (16.0.10358.20061) to determine statistical significance. Kaplan–Meier analysis was performed using GraphPad Prism8 software version 8.4.3 (GraphPad Software, USA) to estimate the survival curve of WT and KO mice, and between-group differences were analyzed with the log-rank test. *p* < 0.05 was considered statistically significant. Significance levels were indicated as follows: *p* < 0.05, ‘*’ *p* < 0.01, ‘**’*p* < 0.001’***’. Additional statistical information, including sample sizes and *p*-values for each experiment, is detailed in the figure legends. Normality tests were not performed because they do not necessarily give reliable results when the sample size is small (n <  ~ 30).

### Supplementary Information

Supplementary Figures.

Supplementary Information.

Supplementary Tables.

## Data Availability

All data generated or analyzed during this study are included in this published article (and its Supplementary Information files). RNA-seq data are available in DDBJ sequencing read archive (DRA) under the accession number DRA016576 (https://ddbj.nig.ac.jp/search).
