# Supplementary material for: Loss of connectin novex-3 leads to heart dysfunction associated with impaired cardiomyocyte proliferation and abnormal nuclear mechanics
Source: Sci Rep. 2024 Jun 14;14:13727. doi: 10.1038/s41598-024-64608-1 (PMC11178842; doi:10.1038/s41598-024-64608-1)
Supplement: Supplementary file 1 — Supplementary Figures. [file 41598_2024_64608_MOESM1_ESM.pdf]

Loss of connectin novex-3 leads to heart dysfunction associated with impaired cardiomyocyte proliferation and abnormal nuclear mechanics.

Ken Hashimoto, Momoko Ohira, Aya Kodama, Misaki Kimoto, Mariko Inoue, Shigenobu Toné, Yuu Usui, Akira Hanashima, Takato Goto, Yuhei Ogura, Yoshihiro Ujihara, Satoshi Mohri

Supplementary Figures S1-S6

Suppl. Fig. 1

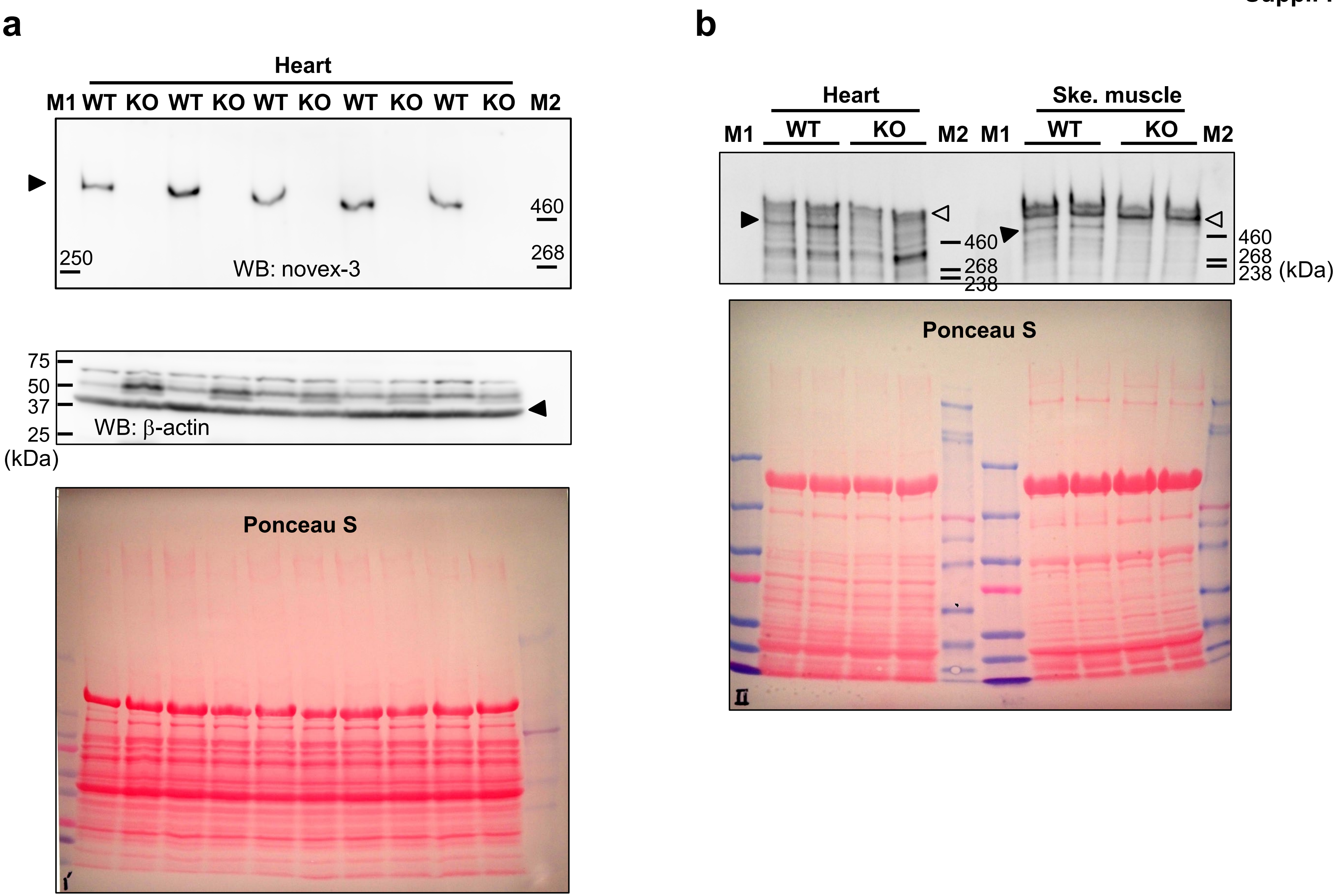
a
Heart
M1 WT KO WT KO WT KO WT KO WT KO M2
460
268
250
WB: novex-3
75
50
37
25
(kDa)
WB: β-actin
Ponceau S
b
Heart
Ske. muscle
M1 WT KO M2 M1 WT KO M2
460
268
238
460
268
238 (kDa)
Ponceau S

c

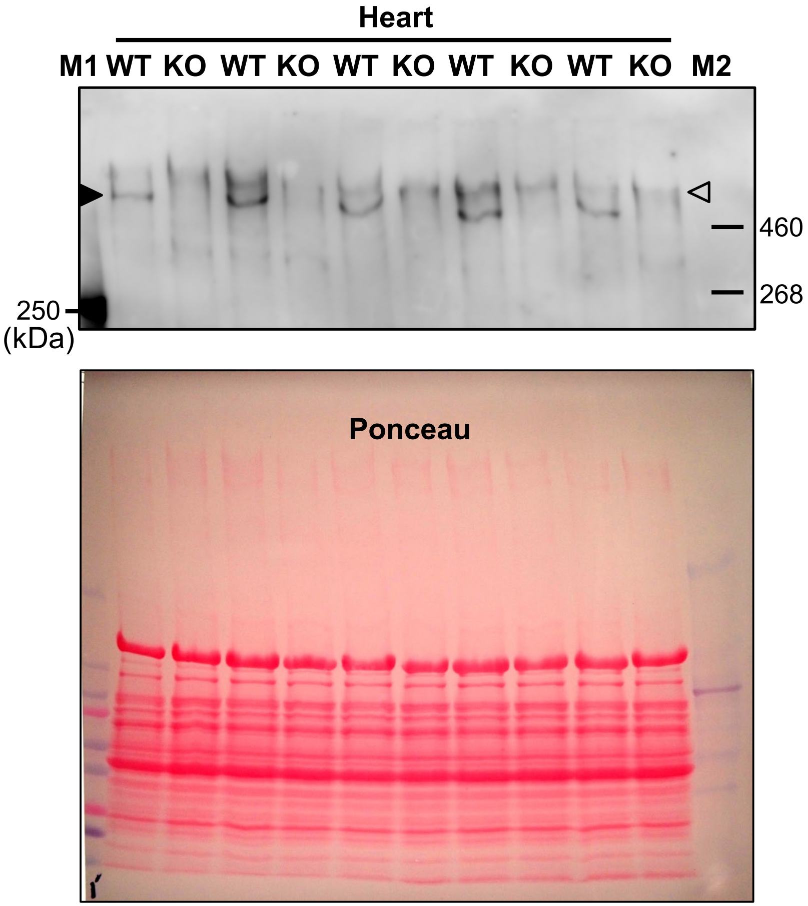

Suppl. Fig. 1

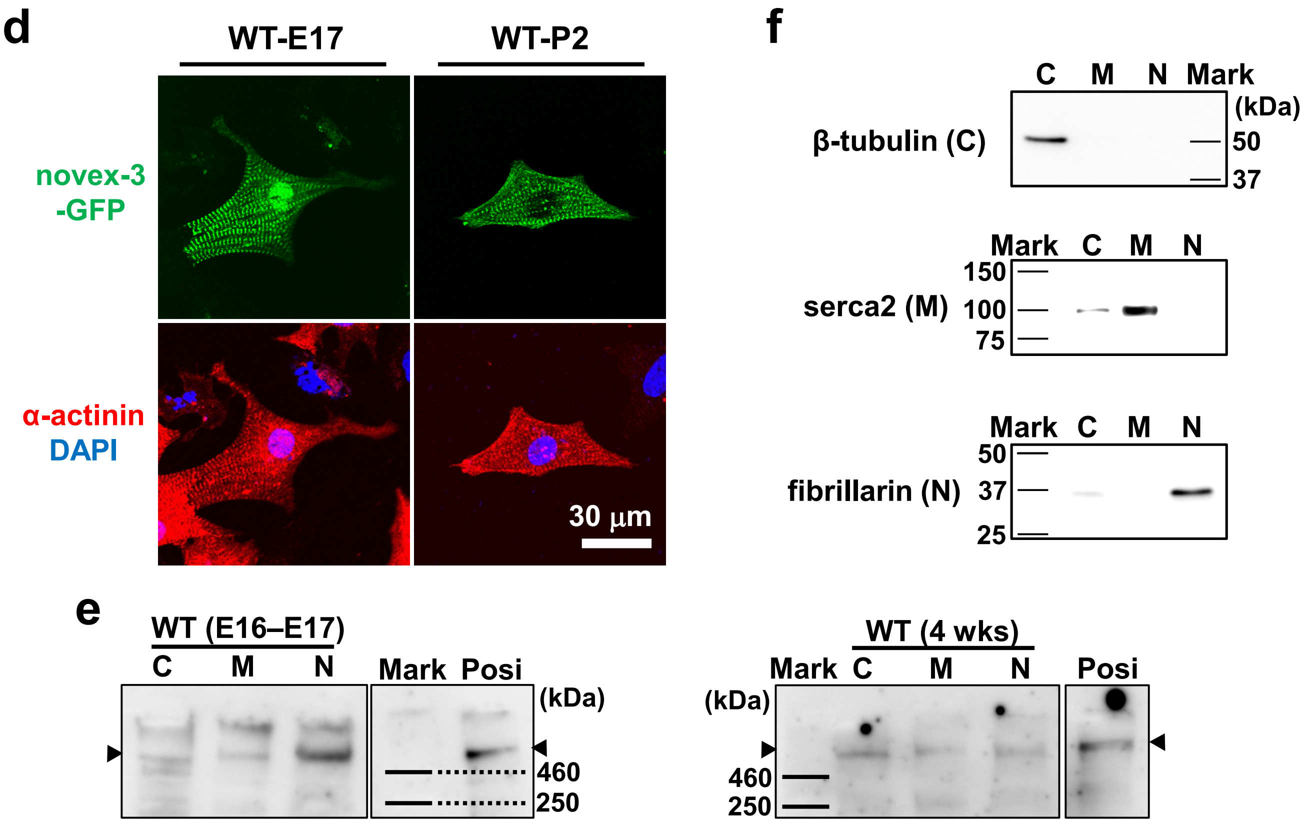

Supplementary Figure 1. The generation of novex-3 KO mice and subcellular localization of novex-3 during development.

a. Western blot for novex-3 proteins (arrowhead) in hearts from WT/KO mice (30–37W) using an antibody against novex-3-specific region (ab1 shown in Fig. 1a). The representative blot for five mice in each condition is shown. The blot for β-actin (marked as arrowhead) and Panceau S staining of the membrane were done as a loading control. M1: size marker (10–250 kDa), M2: size marker (30–460 kDa). **b, c.** Western blot using an antibody against N-terminal shared connectin region (ab3 in Fig. 1a) for detecting both novex-3 (filled arrowhead) and full-length major connectin (open arrowhead) on the same blot in hearts/skeletal muscles (**b**) or hearts (**c**) from WT/KO mice (15–37W). The representative blot for two (**b**) or five (**c**) mice in each condition is shown. Panceau S staining of the membrane was done as a loading control. M1: size marker (10–250 kDa), M2: size marker (30–460 kDa). **d.** Dissociated CMs from WT mice transduced with baculovirus expressing full length novex-3-GFP fusion protein costained with sarcomeric α-actinin (as a CM marker) and DAPI at the indicated age. **e.** Western blot analysis of novex-3 proteins (arrowhead) in hearts from WT mice at the indicated age using an antibody against novex-3-specific region (ab1 in Fig. 1a). C: cytoplasmic fraction, M: membrane fraction, N: nuclear fraction, Posi: novex-3-positive band (arrowhead) from whole heart homogenates. Mark: size marker (30–460 kDa). The separated column between the lane represents the different samples from the same blot. **f.** The fractionation method used in (**e**) was validated by immunoblotting a specific protein with known localization: β-tubulin (C), serca2 (M), and fibrillarin (N). Mark: size marker. (10–250 kDa). In western analysis, original blots are presented in Supplementary Fig. 6.

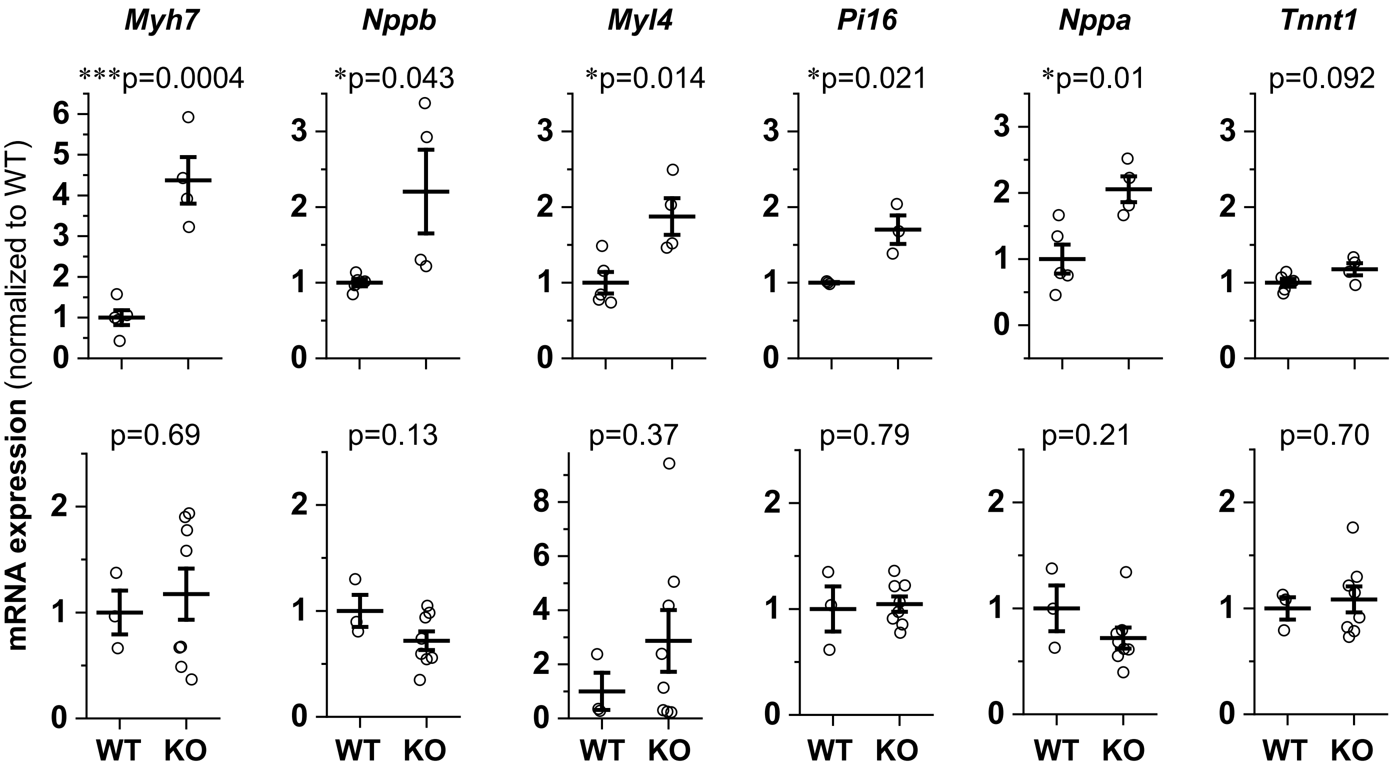

Supplementary Figure 2. Increased expressions of immature undifferentiated marker genes in P1, but not in adult KO hearts. qPCR analysis of immature undifferentiated marker transcripts in P1 (top row) and adult (31–70 wks, bottom row) hearts from WT/KO mice. Data are shown as normalized to WT. n = 3–8 mice per group. * $p < 0.05$ and *** $p < 0.001$ as compared to WT by Student's two-tailed unpaired t-test. Error bar = SEM.

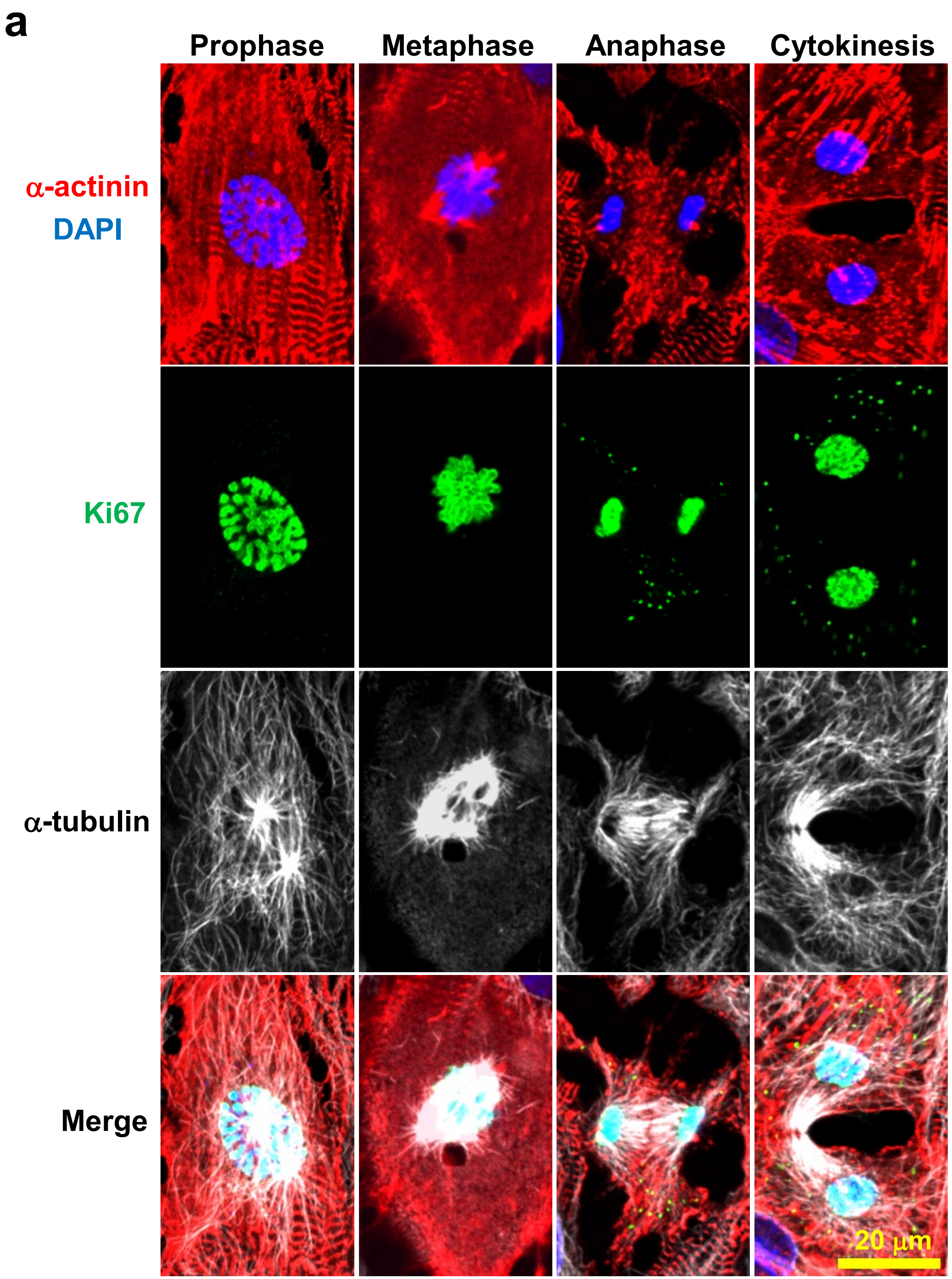

Supplementary Figure 3. Kinetics of microtubules and centrosomal proteins during mitosis and cytokinesis.

Immunofluorescence of microtubule component α-tubulin (**a**) and centrosomal proteins PCM-1 (**b**), PCNT (**c**), and γ-tubulin (**d**) costained with cell cycle marker Ki67, CM marker (α-actinin or cardiac troponin I; cTnI) and DAPI in dissociated E12–E13 CMs. Intracellular distributions of each target protein during mitosis and cytokinesis are shown. The arrow in (**d**) depicts a non-specific signal.

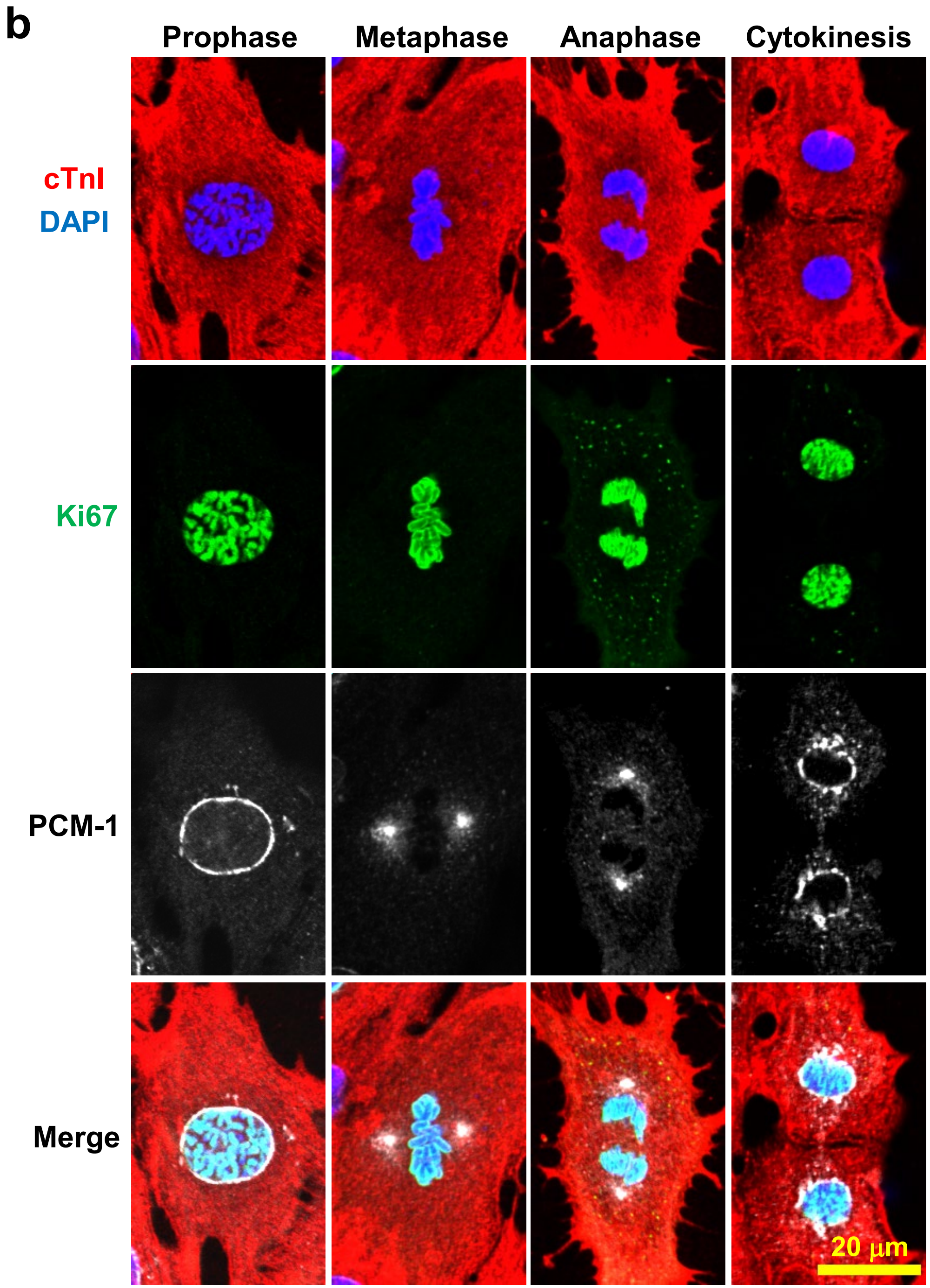
b
Prophase
Metaphase
Anaphase
Cytokinesis
cTnI
DAPI
Ki67
PCM-1
Merge
20 μm

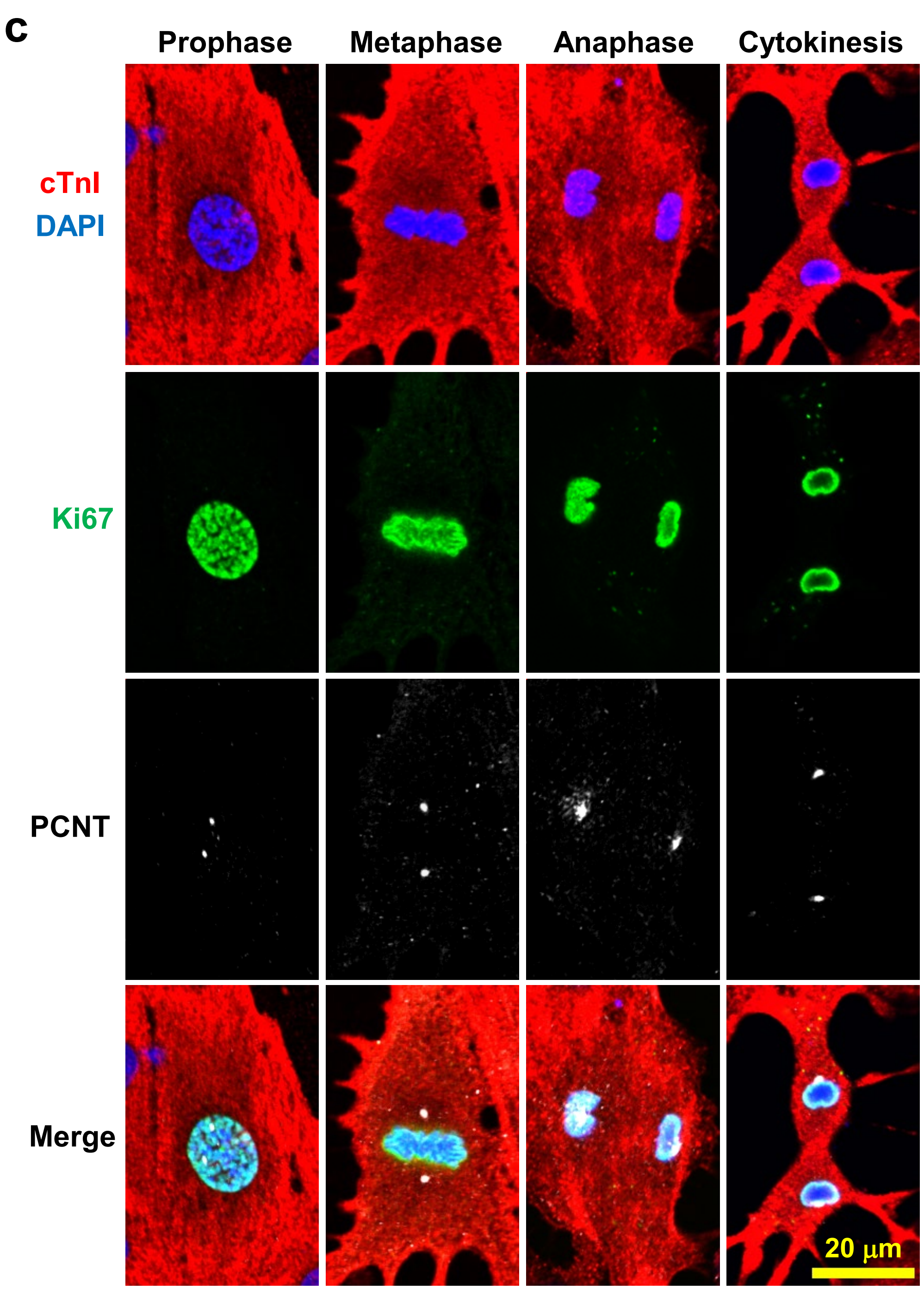
c
Prophase
Metaphase
Anaphase
Cytokinesis
cTnI
DAPI
Ki67
PCNT
Merge
20 μm

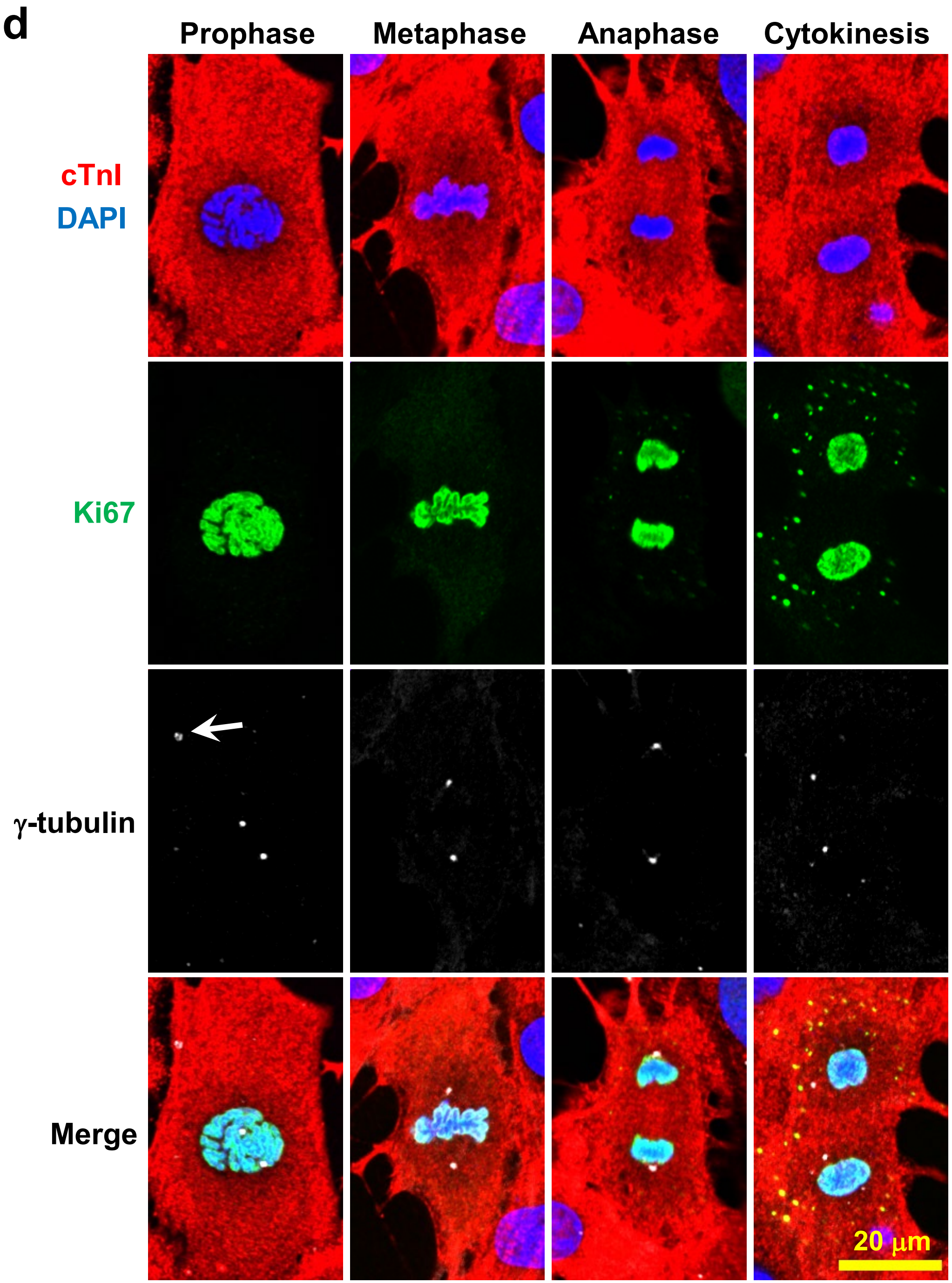
d
Prophase
Metaphase
Anaphase
Cytokinesis
cTnI
DAPI
Ki67
γ-tubulin
Merge
20 μm

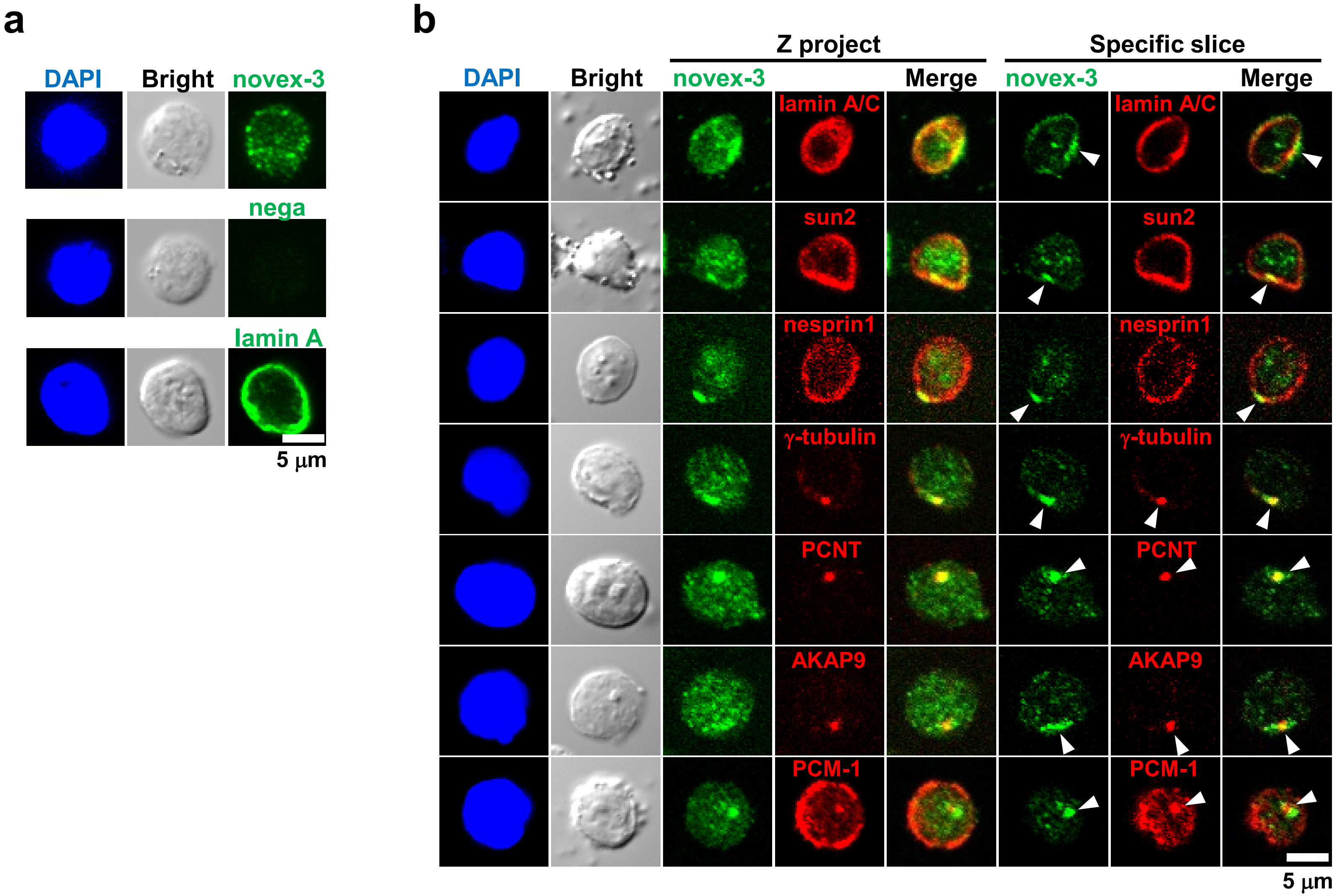

Supplementary Figure 4. Subnuclear localization of novex-3 and its colocalization with nuclear and centrosomal proteins. a. Isolated E17–E18 CM nuclei from WT mice stained with novex-3. nega: no primary antibody control. The integrity of the isolated nuclei was verified by staining patterns of lamin A, DAPI, and bright field image. **b.** Isolated E17–E18 CM nuclei from WT mice double-stained with novex-3 and nuclear membrane proteins (lamin A/C, sun2, and nesprin1) or centrosomal proteins (γ-tubulin, PCNT, AKAP9, and PCM-1) observed in DAPI and bright field image. Z-projected image and the image for the specific slice are shown. Arrowhead: a bright structure of novex-3.

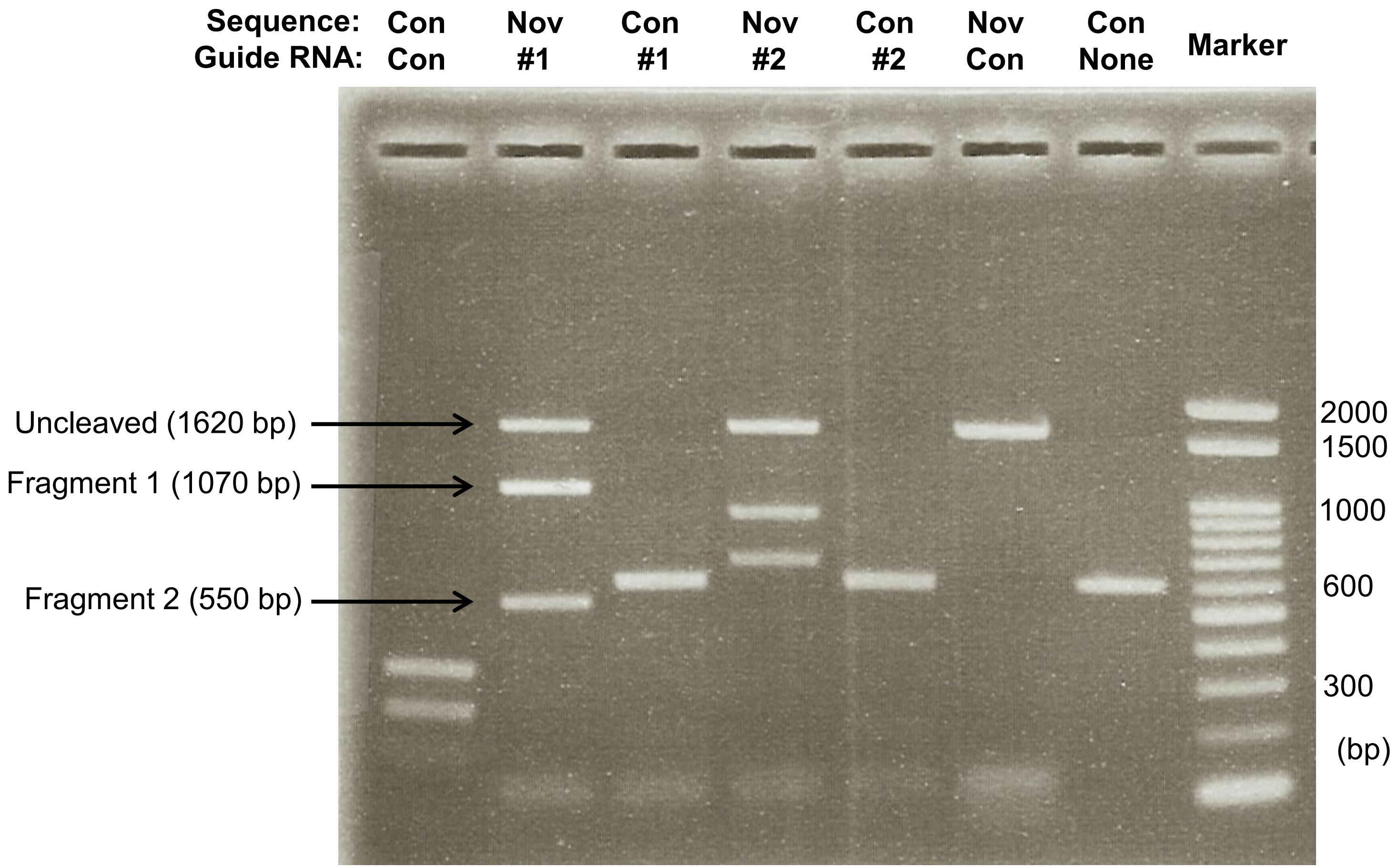

Supplementary Figure 5. In vitro cleavage of the target DNA sequence by candidate guide RNAs.
The two candidate guide RNAs (#1: 5'-TCTCGGTTCTTACGACCCGACGG-3', #2: 5'-CGGCTTGAAATATTCAGGCCAGG-3', PAM sequence underlined) were assessed for in vitro cleavage of the target DNA sequence using Guide-it sgRNA In Vitro Transcription and Screening System (Takara Bio Japan) as per the manufacture's protocol. The target DNA sequence of novex-3 (Nov, 1620 bp) was predicted to be cleaved into two fragments (1070 bp and 550 bp) by the guide RNA #1, and into another two fragments (920 bp and 710 bp) by the guide RNA #2. The positive control DNA sequence (Con, 614 bp) was predicted to be cleaved into two fragments (350 bp and 264 bp) by the positive control guide RNA (Con).

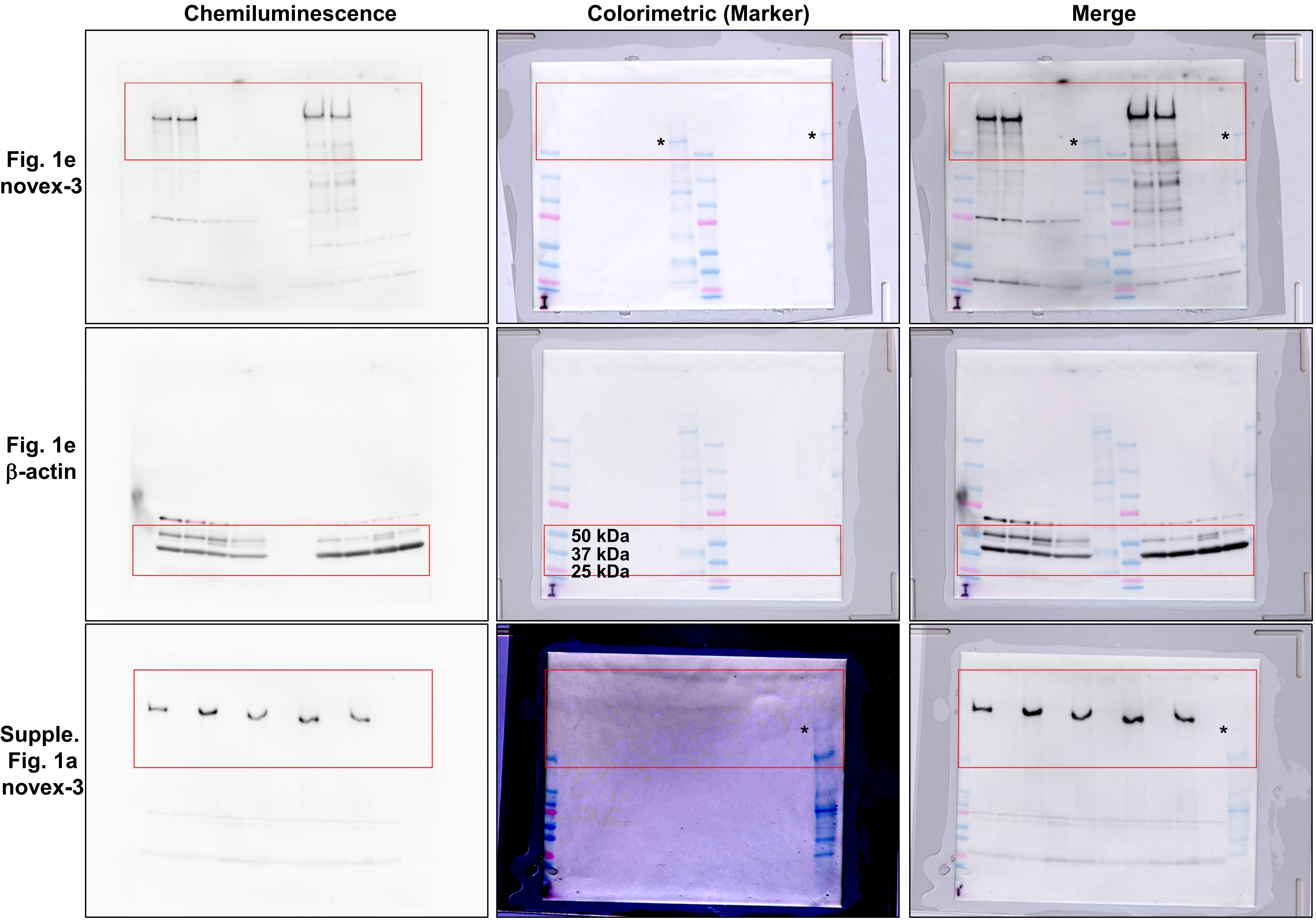

Supplementary Figure 6. Original western blot images.
Each image (chemiluminescence and colorimetric) was captured in the same fixed camera position. Asterisk: 460 kDa.

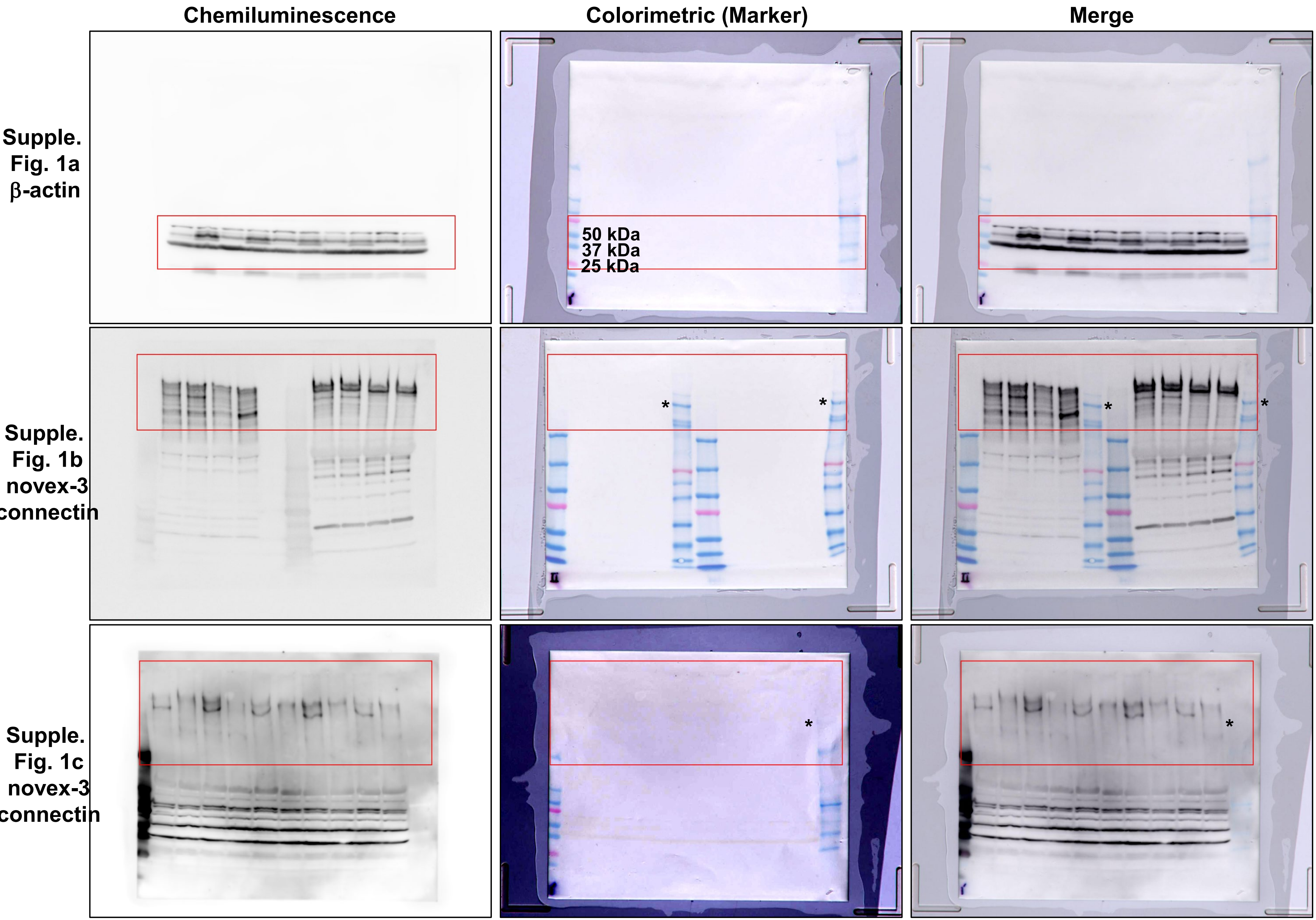

Supplementary Figure 6. Original western blot images.
Each image (chemiluminescence and colorimetric) was captured in the same fixed camera position. Asterisk: 460 kDa

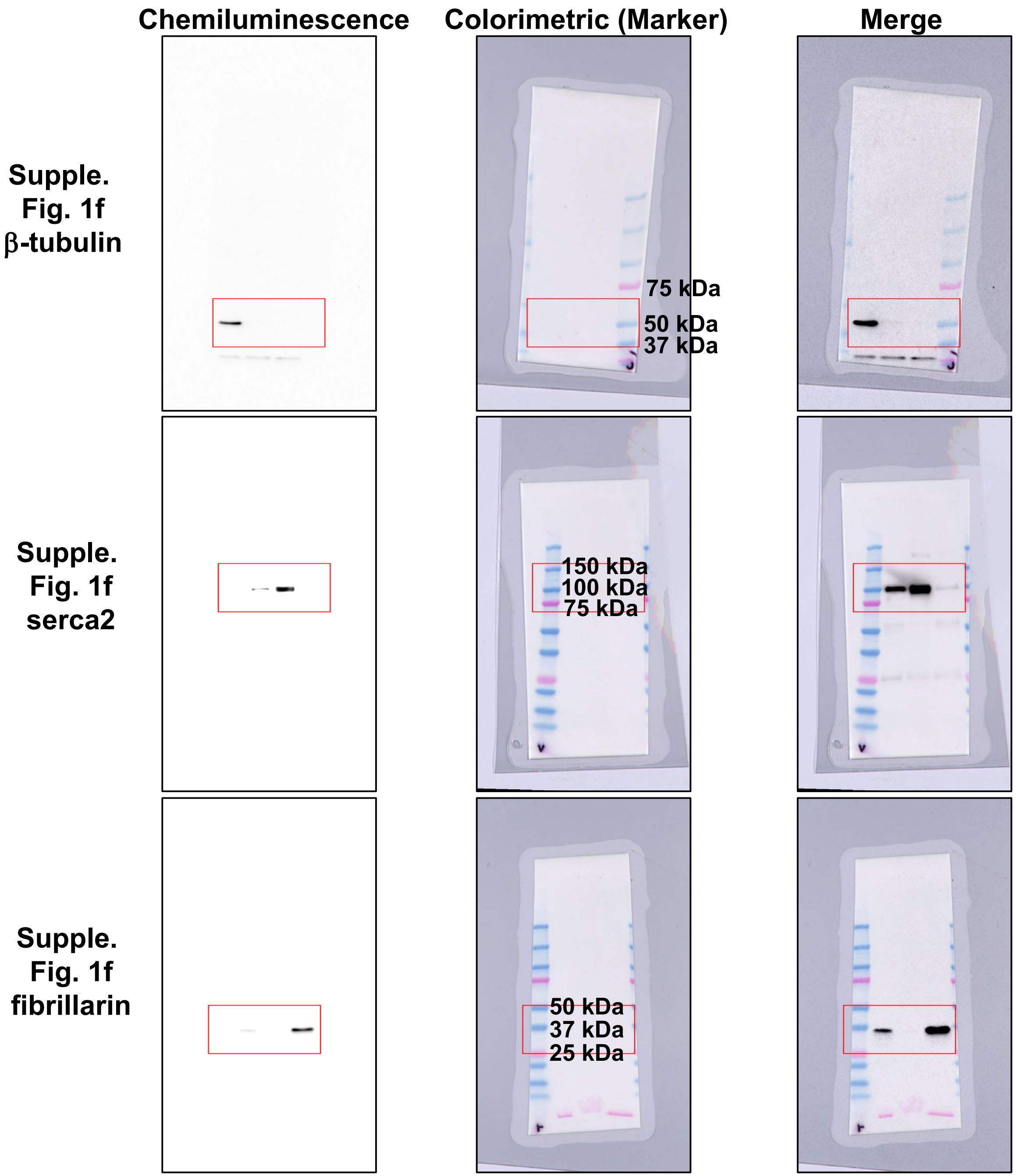

Supplementary Figure 6. Original western blot images.

Each image (chemiluminescence and colorimetric) was captured in the same fixed camera position.

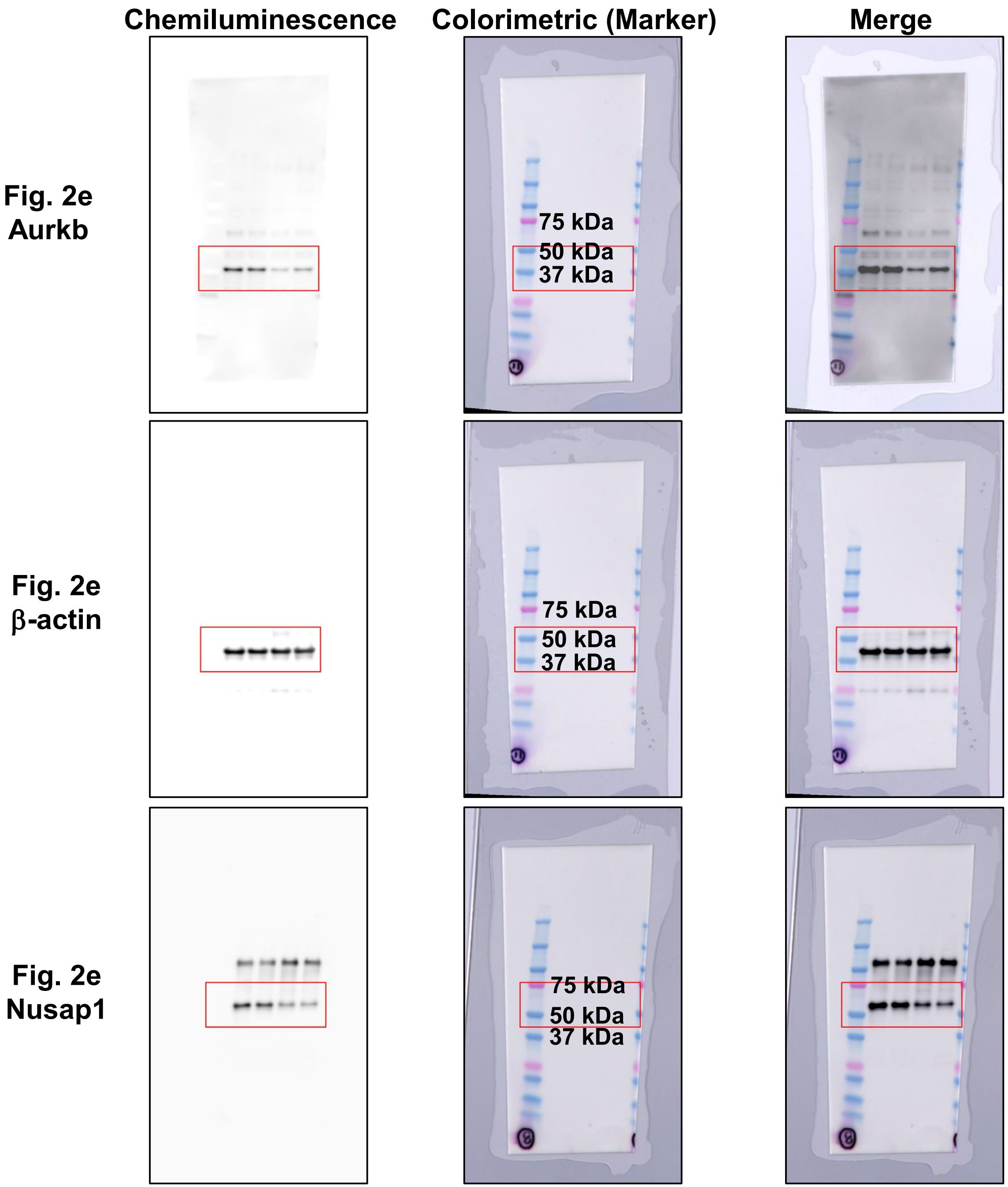

Supplementary Figure 6. Original western blot images.
Each image (chemiluminescence and colorimetric) was captured in the same fixed camera position.

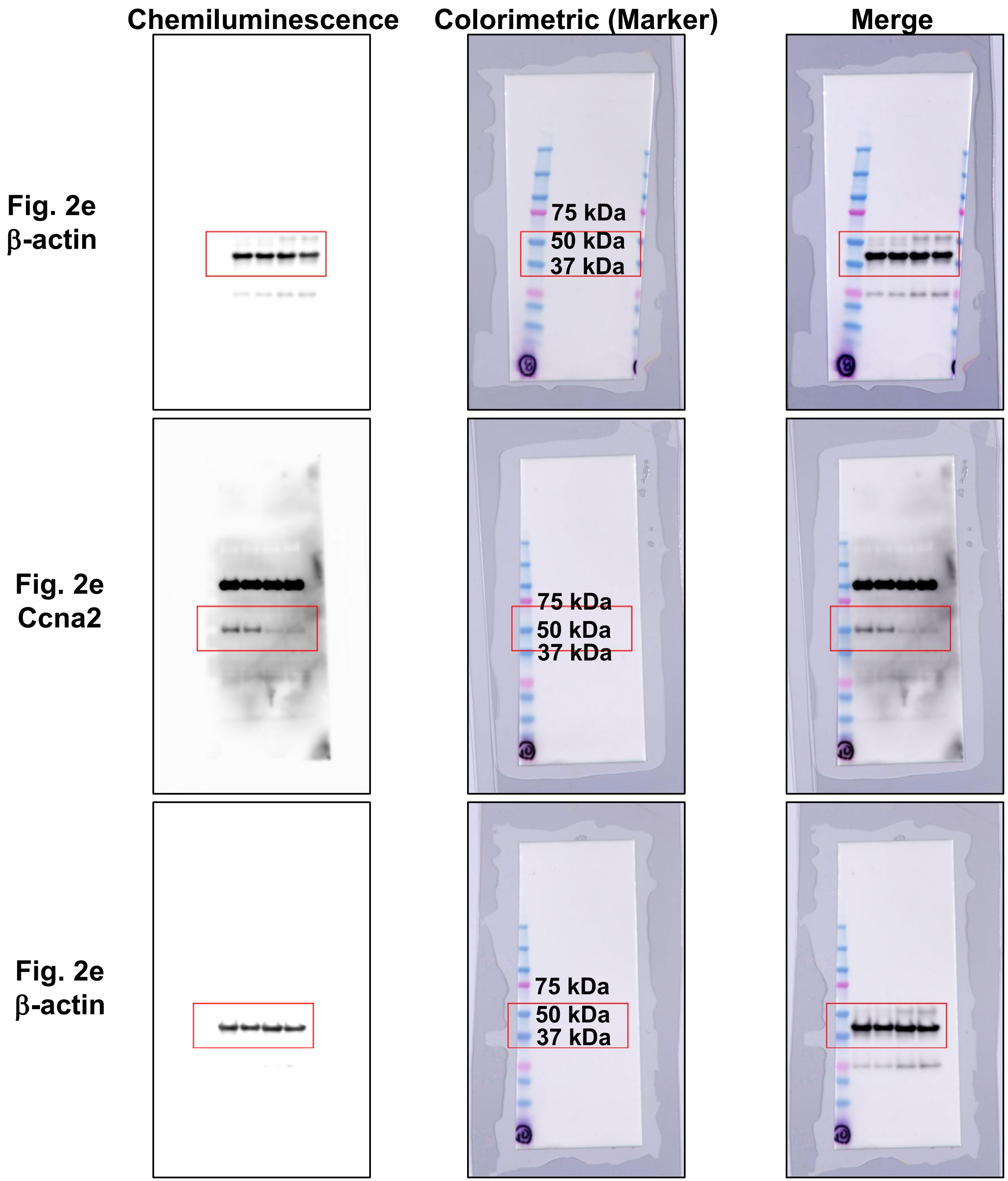

Supplementary Figure 6. Original western blot images.

Each image (chemiluminescence and colorimetric) was captured in the same fixed camera position.

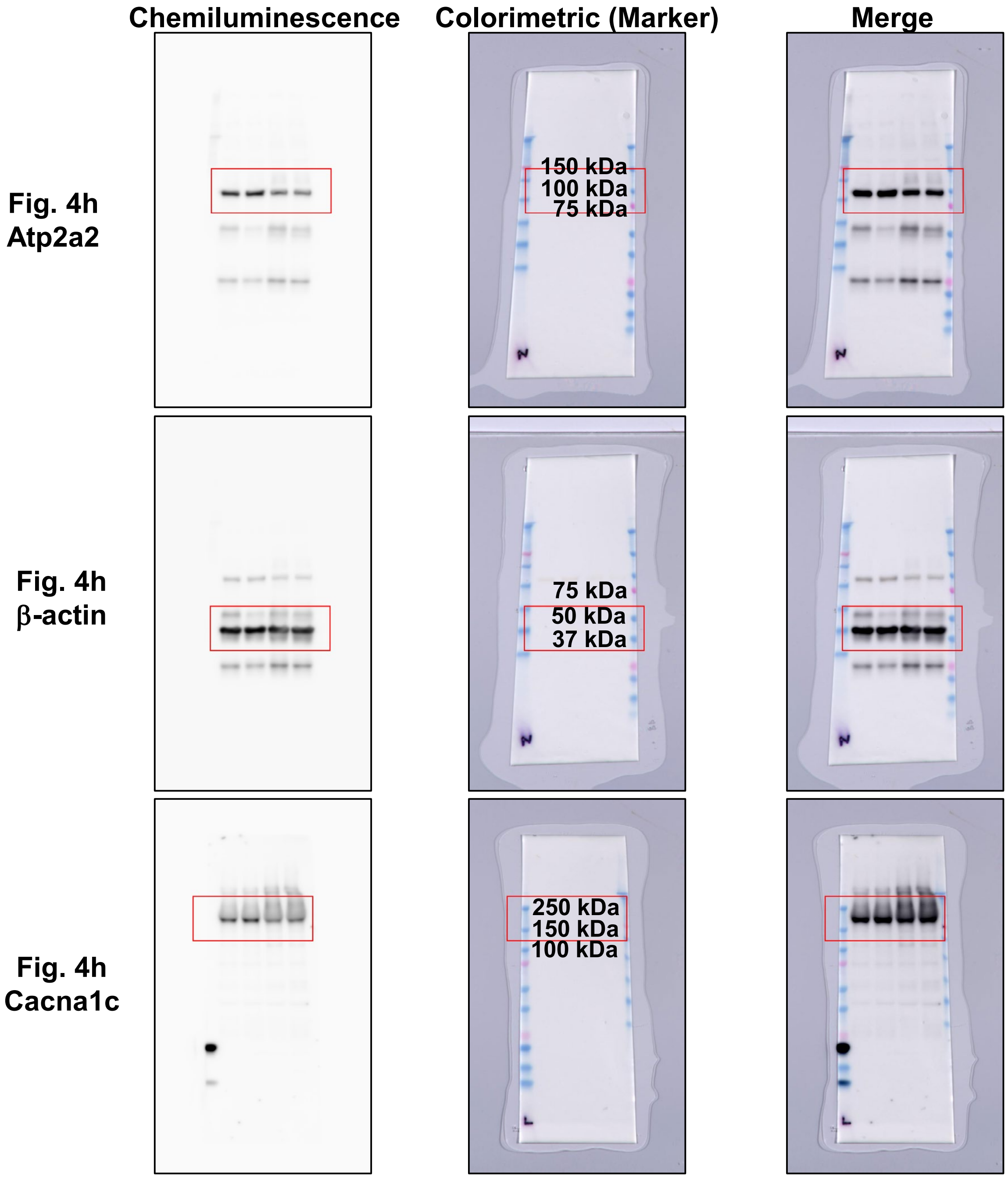

Supplementary Figure 6. Original western blot images.
Each image (chemiluminescence and colorimetric) was captured in the same fixed camera position.

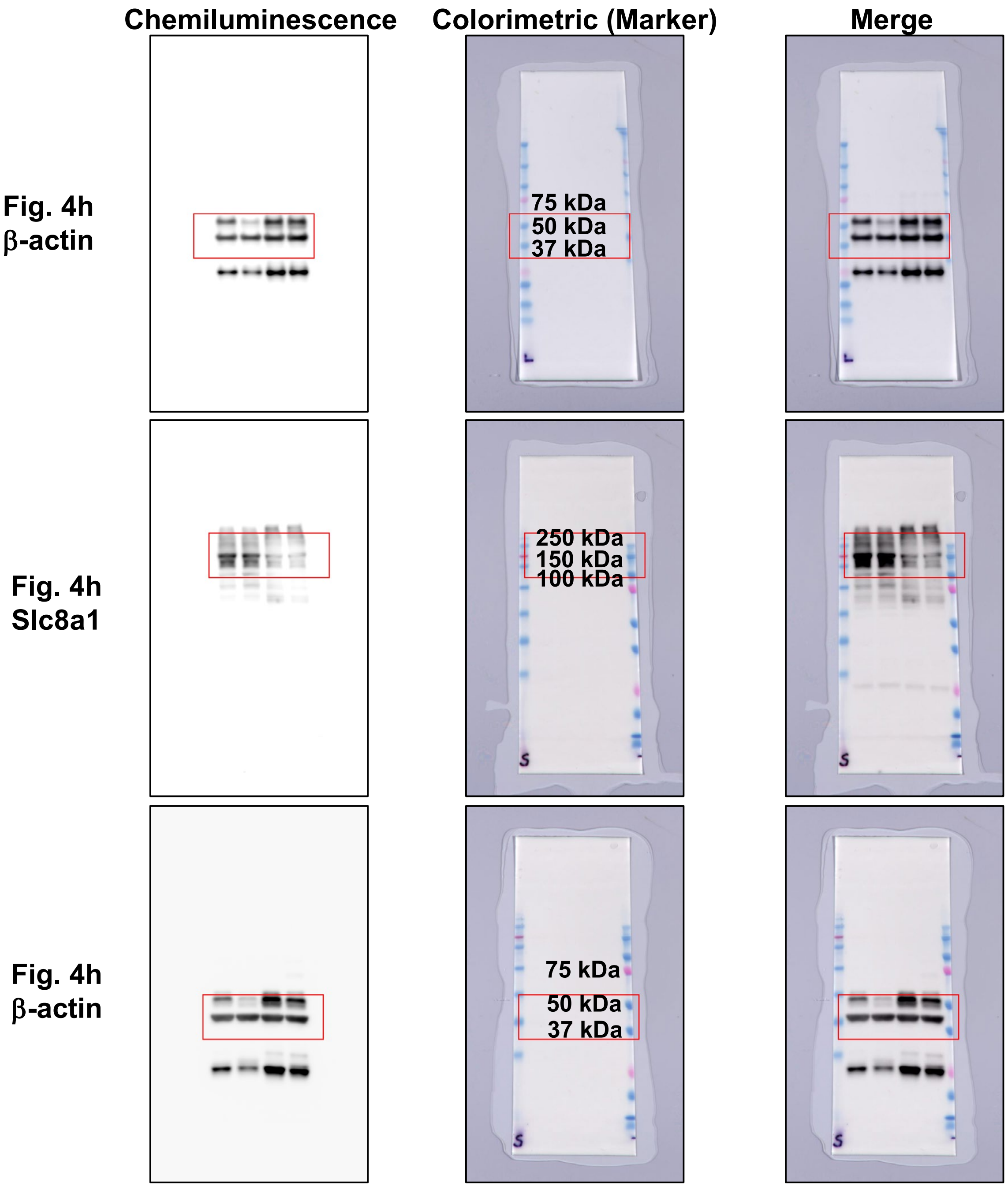

Supplementary Figure 6. Original western blot images.

Each image (chemiluminescence and colorimetric) was captured in the same fixed camera position.

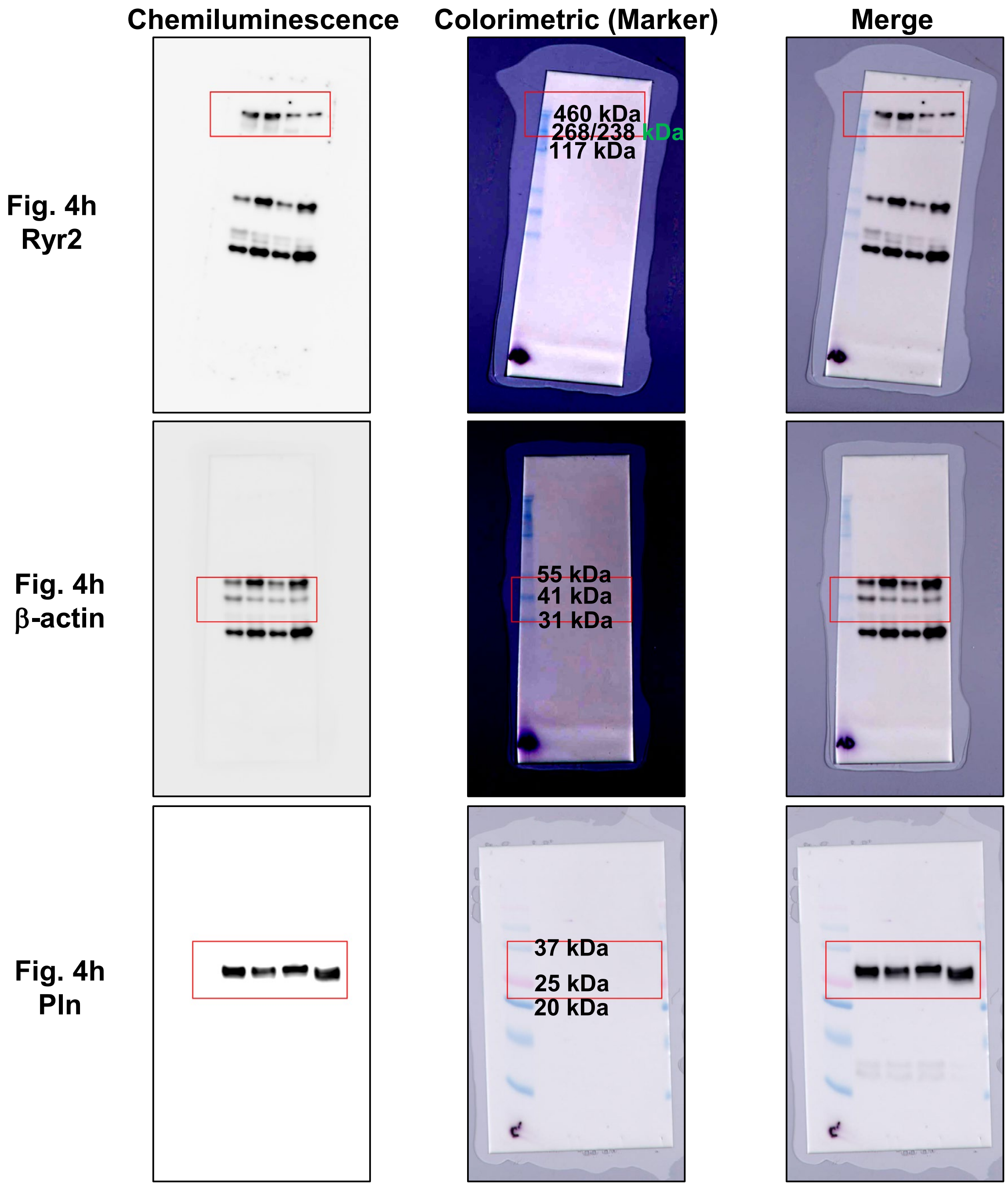

Supplementary Figure 6. Original western blot images.

Each image (chemiluminescence and colorimetric) was captured in the same fixed camera position.

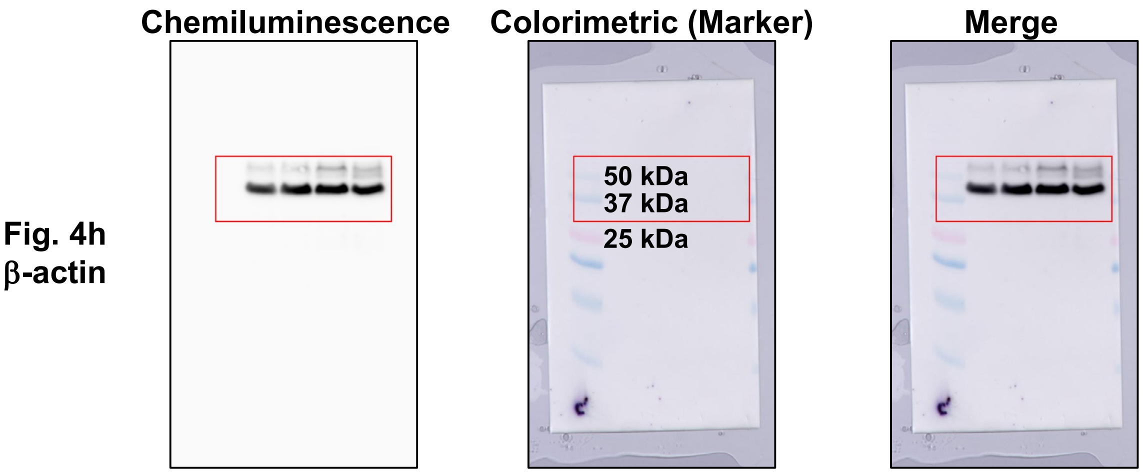

Supplementary Figure 6. Original western blot images.
Each image (chemiluminescence and colorimetric) was captured in the same fixed camera position.

Supple.
Fig. 1e
E16-17

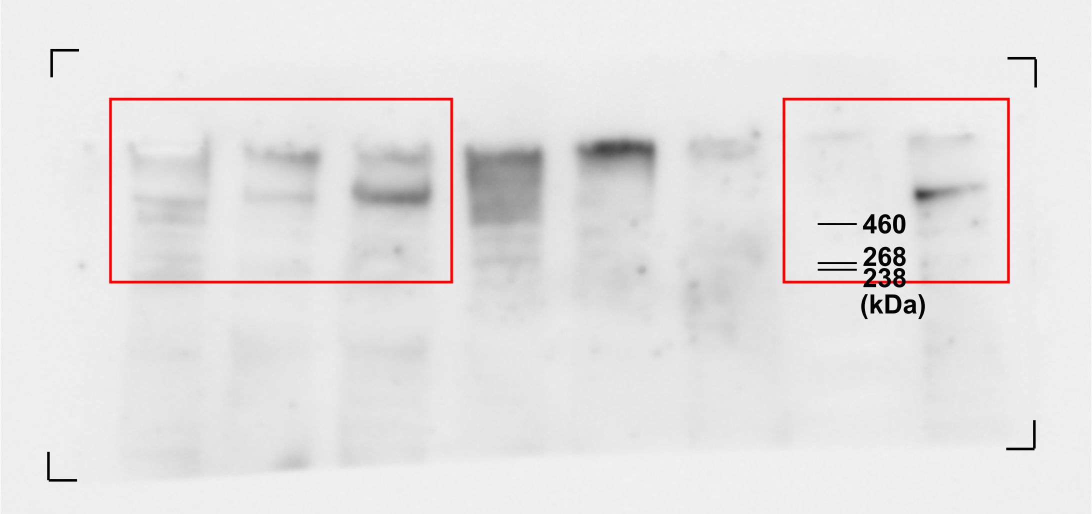

Supple.
Fig. 1e
4 wks

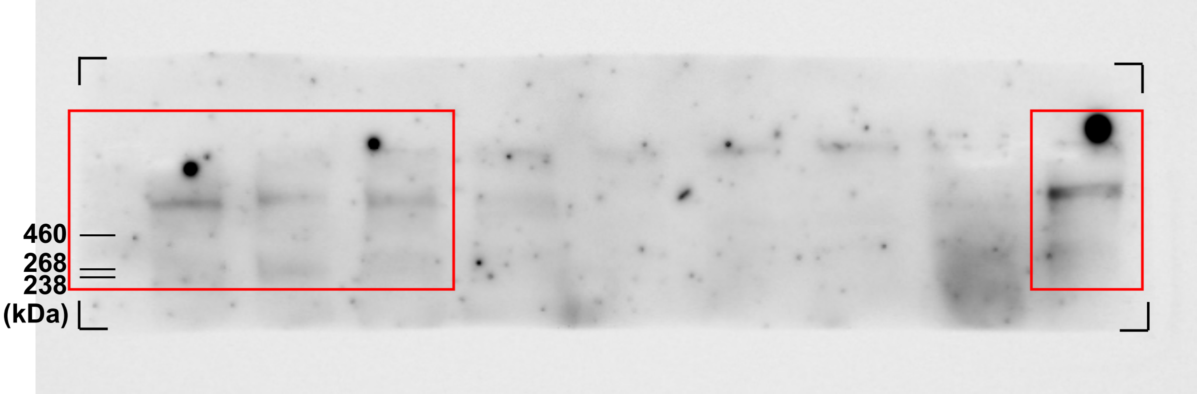

Supplementary Figure 6. Original western blot images.
The edge of the blot is shown as the right-angled black line.
